# WERF Endometriosis Phenome and Biobanking Harmonisation Project for Experimental Models in Endometriosis Research (EPHect-EM-Homologous): homologous rodent models

**DOI:** 10.1093/molehr/gaaf021

**Published:** 2025-07-09

**Authors:** Katherine A Burns, Daniëlle Peterse, Caroline B Appleyard, Ronald Chandler, Sun-Wei Guo, Amelia Pearson, Eleonora Persoons, Michael S Anglesio, Michael S Rogers, Kathy L Sharpe-Timms, Joris Vriens, Stacy L McAllister, Kelsi N Dodds, Fiona L Cousins, Lone Hummelshoj, Stacey A Missmer, Kaylon L Bruner-Tran, Erin Greaves, EPHect Experimental Models Working Group, Nick A Andrews, Michael S Anglesio, Caroline B Appleyard, Joe Arosh, Christian M Becker, Kaylon L Bruner-Tran, Katherine A Burns, Ronald L Chandler, Julie A Christianson, Fiona L Cousins, Kelsi N Dodds, Victor Fattori, Asgi Fazleabas, Caroline Gargett, Juan S Gnecco, Raul Gomez, Martin Götte, Erin Greaves, Linda G Griffith, Patrick G Groothuis, Ruth Grümmer, Sun-Wei Guo, Shannon M Hawkins, M Louise Hull, Lone Hummelshoj, Mark Hutchinson, Mohamed Gamal Ibrahim, Elizabeth E Marr, Stacy L McAllister, Stacey A Missmer, Jeffrey Mogill, Jens Nagel, Warren B Nothnick, Paulina Nunez-Badinez, Kevin G Osteen, Daniëlle Peterse, Michael S Rogers, Andrea Romano, Philippa T K Saunders, Miguel Ángel Tejada, Kathy L Sharpe-Timms, Waldiceu A Verri, Paola Viganó, Katy Vincent

**Affiliations:** Department of Environmental and Public Health Sciences, University of Cincinnati College of Medicine, Cincinnati, OH, USA; Department of Surgery, Vascular Biology Program, Boston Children’s Hospital, Harvard Medical School, Boston, MA, USA; Department of Basic Sciences, Ponce Health Sciences University—Ponce Research Institute, Ponce, PR, USA; Department of Obstetrics, Gynecology and Reproductive Biology, College of Human Medicine, Michigan State University, Grand Rapids, MI, USA; Research Institute, Shanghai Obstetrics and Gynecology Hospital, Fudan University, Shanghai, China; Department of Environmental and Public Health Sciences, University of Cincinnati College of Medicine, Cincinnati, OH, USA; Department of Development and Regeneration, University of Leuven, Leuven, Belgium; Department of Obstetrics and Gynaecology, University of British Columbia, Vancouver, BC, Canada; Department of Surgery, Vascular Biology Program, Boston Children’s Hospital, Harvard Medical School, Boston, MA, USA; Department of Obstetrics, Gynecology and Women’s Health, The University of Missouri School of Medicine, Columbia, MO, USA; Department of Development and Regeneration, University of Leuven, Leuven, Belgium; Department of Gynecology and Obstetrics, Emory School of Medicine, Atlanta, GA, USA; College of Medicine and Public Health and Flinders Health and Medical Research Institute, Flinders University, Bedford Park, SA, Australia; School of Biomedicine and Institute for Photonics and Advanced Sensing, University of Adelaide, Adelaide, SA, Australia; The Ritchie Centre, Hudson Institute of Medical Research, Clayton, VIC, Australia; Department of Obstetrics and Gynaecology, Monash University, Clayton, VIC, Australia; World Endometriosis Research Foundation, London, UK; World Endometriosis Research Foundation, London, UK; Department of Epidemiology, Harvard TH Chan School of Public Health, Boston, MA, USA; Department of Obstetrics and Gynecology, University of Michigan, Ann Arbour, MI, USA; World Endometriosis Research Foundation, London, UK; Department of Obstetrics and Gynecology, Vanderbilt University Medical Center, Nashville, TN, USA; World Endometriosis Research Foundation, London, UK; Division of Biomedical Sciences, Warwick Medical School, University of Warwick, Coventry, UK

**Keywords:** endometriosis, experimental models, rodents, homologous, research, collaboration

## Abstract

*In vivo* models of endometriosis enable the discovery and preclinical testing of new therapies. Several rodent models of endometriosis exist, but a lack of harmonization impedes reproducibility and comparability of results among investigators. Homologous models are advantageous as they allow the contribution of the immune system/inflammation to be studied. We reviewed published homologous rodent models of endometriosis to develop standard operating procedures (‘EPHect-EM-Homologous-SOPs’) to guide and facilitate the choice and implementation of these models and harmonize documentation to enhance interpretation and comparability of results. The World Endometriosis Research Foundation (WERF) established an international working group of experts in models of endometriosis and formed a working sub-group to discuss homologous rodent models of endometriosis. A systematic literature review and detailed analysis of protocols was performed. The identified models have advantages and limitations regarding physiological relevance and utility. To harmonize key variables for endometriosis rodent models, the working group focused on species and animal strains, placement of ectopic tissue, uterine tissue volume, method of induction, hormonal status, and uterine tissue ‘type’. A decision tree and recommendations on model use were developed for mice and rats to serve as guides for the use of harmonized EPHect-EM-Homologous-SOPs, experimental design, reporting standards, and research of question-dependent key variables. No ‘ideal’ homologous model of endometriosis was identified. The choice of model for specific research should be guided according to a best-fit strategy. Harmonization of SOPs, documentation, and reporting standards will improve replicability and translational applicability of studies and better highlight where *de novo* model creation is needed.

## Introduction

Endometriosis affects an estimated 190 million women (and those assigned female at birth) worldwide with a significant personal and societal burden due to its painful and fertility-related symptoms (Nnoaham *et al.*, 2011; Zondervan *et al.*, 2020). There is no known cure, and treatments are associated with low long-term success rates and significant side effects (Johnson *et al.*, 2013).

Endometriosis is defined as endometrial-like tissue outside the uterus; however, this definition does not encompass the complex symptomatic, pathobiological, and multisystemic nature of the disease (Zondervan *et al.*, 2020). The overwhelming prevalence of endometriosis and lack of known aetiology highlights the need for a better understanding of the basic biology of endometriosis formation and persistence to enable subsequent identification of targets for effective therapies for endometriosis.

Endometriosis lesions are complex multicellular tissue deposits composed of endometrial-like stromal cells, epithelial glands, and extracellular matrix (ECM) deposition including fibrosis and scarring, sometimes with evidence of haemorrhage (Clement, 2007; Vigano *et al.*, 2018). They are infiltrated by blood vessels and nerve fibres as well as abundant immune cells, yielding a highly inflammatory microenvironment (Burns *et al.*, 2012, 2018; Greaves *et al.*, 2017a; Forster *et al.*, 2019; Hogg *et al.*, 2020; Hogg *et al.*, 2021; Panir *et al.*, 2022; Fattori *et al.*, 2024). The local cellular niche of a lesion is regulated by oestrogen, which has significant impacts on endometrial cell types (Saunders and Horne, 2021) as well as processes such as neuroangiogenesis and neuroinflammation (Greaves *et al*., 2014a, 2015). The histological appearance of lesions varies significantly, with lesions having different contributions of ECM, fibrosis (Li *et al.*, 2022) and endometrial-like cells (Clement, 2007) and differences in their synchronicity with the menstrual cycle (Colgrave *et al.*, 2020) as well as the extent of immune and nerve infiltration (Tran *et al.*, 2009). It is likely that disease processes vary in each lesion, as suggested by the extreme variation in disease lesion subtype, symptom presentation, and response to treatment, as well as the hypothesized ‘life-cycle’ of lesions that progress through different stages and colours (Zondervan *et al.*, 2020).

Lesions are currently classified into three major subtypes: (i) superficial peritoneal, (ii) ovarian endometriosis (endometriomas), and (iii) deep lesions (International Working Group of AAGL, ESGE, ESHRE and WES *et al.*, 2021). Associated genomic loci vary by lesion subtype, with most of the 42 loci identified to date associated with endometriomas (Rahmioglu *et al.*, 2023). Researchers now understand more about the pathobiology of endometriosis than ever before; however, the exact aetiology of the disease remains elusive and may vary between patients and lesion subtypes.

As with most conditions with known genetic and phenotypic complexity, current evidence confirms that there is unlikely to be a unified theory for endometriosis that explains the different lesion subtypes while also integrating epigenetic, genetic, and immune aberrations and postulated environmental exposures that are thought to contribute to the disease (Zondervan *et al.*, 2018; Giudice *et al.*, 2023). Instead, the different lesion subtypes may represent different disease entities, with possible divergent origins (Nirgianakis *et al.*, 2020; Imperiale *et al.*, 2023; Rahmioglu *et al.*, 2023). The most commonly invoked theory of aetiology is Sampson’s theory of retrograde menstruation, which states that menstrual effluent is disseminated into the peritoneal cavity via the fallopian tubes, where it migrates, attaches, grows, and invades peritoneal tissues as endometriotic lesions (Sampson, 1927a,b). However, Sampson’s theory is insufficient, as only ∼10% of reproductive-age women suffer from endometriosis (Halme *et al.*, 1984), while most women, both with and without endometriosis, exhibit some levels of retrograde menstruation (Halme *et al.*, 1984; Bartosik *et al.*, 1986; Kruitwagen *et al.*, 1991; Sharpe-Timms, 2005; O *et al.*, 2017). Nonetheless, a uterine origin provides a simple and attractive source for the cell types involved, and somatic genetic data may favour this aetiology (Anglesio *et al.*, 2017; Suda *et al.*, 2018; Lac *et al.*, 2019a; Moore *et al.*, 2020; Praetorius *et al.*, 2022; Yamaguchi *et al.*, 2022). Indeed, there are examples of clonality between uterine epithelium and endometriotic lesion epithelium (Inoue *et al.*, 2019; Bulun *et al.*, 2023). Additionally, these mutations may contribute to fibrogenesis in lesions (Guo, 2018a) that selects for clones bearing these mutations. Other theories encompassing coelomic metaplasia, vascular and lymphatic spread, stem cells (and neonatal bleeding), Müllerian remnants (Vigano *et al.*, 2018), and a combination of theories may explain at least some of the different forms/occurrences of endometriotic lesions (Tsamantioti and Mahdy, 2022). Other contributing factors that have been identified recently are not restricted to any one theory of origin and include heritable features, immune microenvironment, somatic mutations, as well as insights provided through epigenomic, metabolomic and proteomic studies, as well as demographic, anthropometric, and/or other personal characteristics. Contributing further to the complexity and enigma of the disease, lesion phenotype and burden do not correlate with clinical symptoms of endometriosis (Litson *et al.*, 2022).

Previously, the World Endometriosis Research Foundation (WERF) Endometriosis Phenome and Biobanking Harmonisation Project (EPHect) established standard recommendations and minimum requirements for the collection of clinical and surgical data from those with endometriosis (Becker *et al.*, 2014; Vitonis *et al.*, 2014), as well as a standardized physical examination assessment in endometriosis (Lin *et al.*, 2024). WERF also developed internationally agreed-upon standard operating procedures (SOPs) for the collection, processing, and storage of tissue and fluid biospecimens (Becker *et al.*, 2014; Fassbender *et al.*, 2014; Rahmioglu *et al.*, 2014; Vitonis *et al.*, 2014). Through this process, WERF identified an unmet need to develop standards of practice in experimental models of endometriosis.

Rodent models of endometriosis are most commonly used for *in vivo* studies. These models are an attractive choice due to the short generation time, relatively low husbandry costs and space requirements, availability of genetically modified strains of mice and rats, and the ability to have large experimental groups (Burns *et al.*, 2021). The first rodent model for endometriosis, describing auto-transplantation of homologous uterine tissues in the rat, was published in 1985 (Vernon and Wilson, 1985). This model, with moderate modifications, has been used widely for studies on the mechanisms of attachment and growth of ectopic tissue and the pathophysiology of endometriosis, including pain and subfertility. Subsequently, one of the first mouse models of endometriosis aimed to replicate the rat model of endometriosis (Cummings and Metcalf, 1995); however, in mice, multiple adaptations have emerged with the aim of improving physiological relevance and refining animal welfare in line with the 3Rs: Replacement, Reduction, and Refinement (Hubrecht and Carter, 2019). Current homologous rodent models that are used to study endometriosis include both rats and mice and range from the surgical engraftment of uterine tissue to injection of ‘menses’-like endometrium (Cummings and Metcalf, 1995; Grummer, 2006; Pelch *et al.*, 2010; Pelch *et al.*, 2012; Wilson *et al.*, 2020; Burns *et al.*, 2021). While each model has strengths and weaknesses and each has served a purpose in helping to understand the pathophysiology of endometriosis, it is important to consider each strength and weakness prior to the design of preclinical studies.

*In vivo* models are required to determine the molecular underpinnings of endometriosis, to develop and test potential therapeutics, and to discover non-invasive biomarkers for disease diagnosis. However, the variety of preclinical models of endometriosis in use causes problems with replicability and direct comparison of study findings. An important concern for all animal research intended for the study of human disease is the translatability of the animal model and whether improvements in preclinical study design can enhance its relevance to human disease (de Caestecker *et al.*, 2015). The process of drug development is complex, lengthy, and expensive; thus, the validity, scientific rigour, and predictive nature of the animal model used are paramount for successful therapeutic development (Groothuis and Guo, 2018). Clinical trials testing potential therapeutics for endometriosis often fail due to lack of efficacy (Guo and Groothuis, 2018), presumably because preclinical testing was not suitably robust or the translatability of the model was limited. Unfortunately, failed or informatively null studies are often not reported or published, even though these findings would inform the field and yield improved understanding of protocols, model validity, and treatments/drugs explored, and would prevent replication of studies that are unlikely to be successful.

A possible concern for translatability in endometriosis research is the recognition that no single rodent model of endometriosis will fully recapitulate the disease among all endometriosis phenotypes for the following reasons; (i) In contrast to humans and some non-human primates, rodents do not develop endometriosis spontaneously (rodents remodel their endometrial lining during the oestrous cycle without endometrial shedding; a notable exception is the spiny mouse (Bellofiore *et al.*, 2017)); (ii) to date, no rodent model of experimental endometriosis reproduces all three main subtypes of endometriotic lesions (though not all humans experience all three subtypes); (iii) there is limited evidence on the true aetiology of endometriosis in women; and (iv) since rodents do not menstruate, the induced lesions likely do not undergo the repeated tissue injury and repair (ReTIAR), as proposed in humans – a process that facilitates lesion progression and fibrogenesis (Zhang *et al.*, 2016a,b; Guo, 2018b). As such, the subsequent model may not recapitulate all the pathways involved in lesion development and progression in humans, nor can it accurately model all details of human endometriosis pathophysiology, such as endometriosis-associated dysmenorrhoea or subfertility.

Acknowledging these hurdles, a working group was established to develop baseline standards for rodent models in endometriosis research and to develop internationally agreed-upon SOPs for experimental models in endometriosis to limit procedural variation and improve reporting standards to maximize inference and comparability without stifling scientific creativity. We also identified gaps in knowledge and offered recommendations for model design and reporting standards, which will allow researchers to compare across studies and laboratories to increase study fidelity, enhance the robustness of preclinical research, and improve the relevance of rodent models to human disease.

Since the complex aetiology of endometriosis is not yet fully understood, our recommendations have endeavoured to ensure the model(s)/SOPs presented maintain sufficient flexibility to not hamper discovery, but to be used to help uncover novel and confirm known aetiology, pathogenesis, and disease progression. In endometriosis research, the main variables in rodent model design are as follows: (i) species and animal strain, (ii) location of ectopic tissue placement, (iii) volume of donor tissue, (iv) method of induction, (v) hormonal status of recipient, and (vi) donor uterine tissue ‘type’. Due to these variations and the increasing number of models and researchers entering the field, WERF initiated this project to develop recommendations for homologous rodent models for endometriosis research.

Recommendations and SOPs were also developed for heterologous rodent models, endometriosis organoids, and pain behaviour, and the results are presented in the associated WERF companion papers (Hull *et al.*, 2025; Marr *et al.*, 2025; and Dodds *et al.*, 2025, respectively).

## Methods

To analyse and assess existing rodent models for their ability to demonstrate the key hallmarks of endometriosis and standardize procedures, including visualization of lesions, pain-associated behavioural measurements, and/or investigation of the subfertility of the animals, internationally renowned endometriosis researchers were invited to join a consortium. Consortium members were identified based on pioneering work with specific rodent model(s) for endometriosis and the criteria of having published manuscripts with (i) evidence of comprehensive histological analysis, (ii) exemplary experimental design, and/or (iii) having published at least three research studies using the same model. For identification of publications, the search criteria were as follows: ‘endometriosis’ + ‘mouse’ + ‘murine’ + ‘rat’. Of the experts invited, 39 accepted. An additional six observers who were students or early career endometriosis researchers were also included. Of the 39 original working group members, 27 and 29, respectively, attended the workshops in June 2021. Eight working group members were added at a later date, and three left the group, leaving a total of 44 contributors. The aims of the workshops were to reappraise the current and emerging models of endometriosis and to develop protocols and criteria (see Supplementary Fig. S1) that meet a minimum set of standards based on the objective(s) of the study. All models were examined with the understanding that an individual model is unlikely to address all parameters of endometriosis, and instead the focus was on identifying research standards critical for the use of rodent models in the study of endometriosis. Following the workshops, consortium members were assigned or self-selected to one or more working groups based on their affinity and expertise (Supplementary Fig. S1). The four groups were: (i) heterologous models, (ii) homologous models, (iii) models to assess pain behaviour, and (iv) organoids as an emerging (*in vitro*) model for endometriosis. For each group, a discussion forum was used to submit SOPs for circulation, standardization, and to enable a platform for discussions. Further discussions were held between authors to continue dialogues and develop consensus on areas for unification.

For the homologous working group, 14 protocols were received from members of the group; these reflected the full spectrum of models described in the literature. A standard for how to format individual protocols was developed. Each investigator added additional requirements to their respective protocol(s) to ensure maximum detail for comparison across all protocols. Next, the protocols were examined for similarities, differences and key areas that could lead to new scientific discovery.

## Results

**Figure 1.**
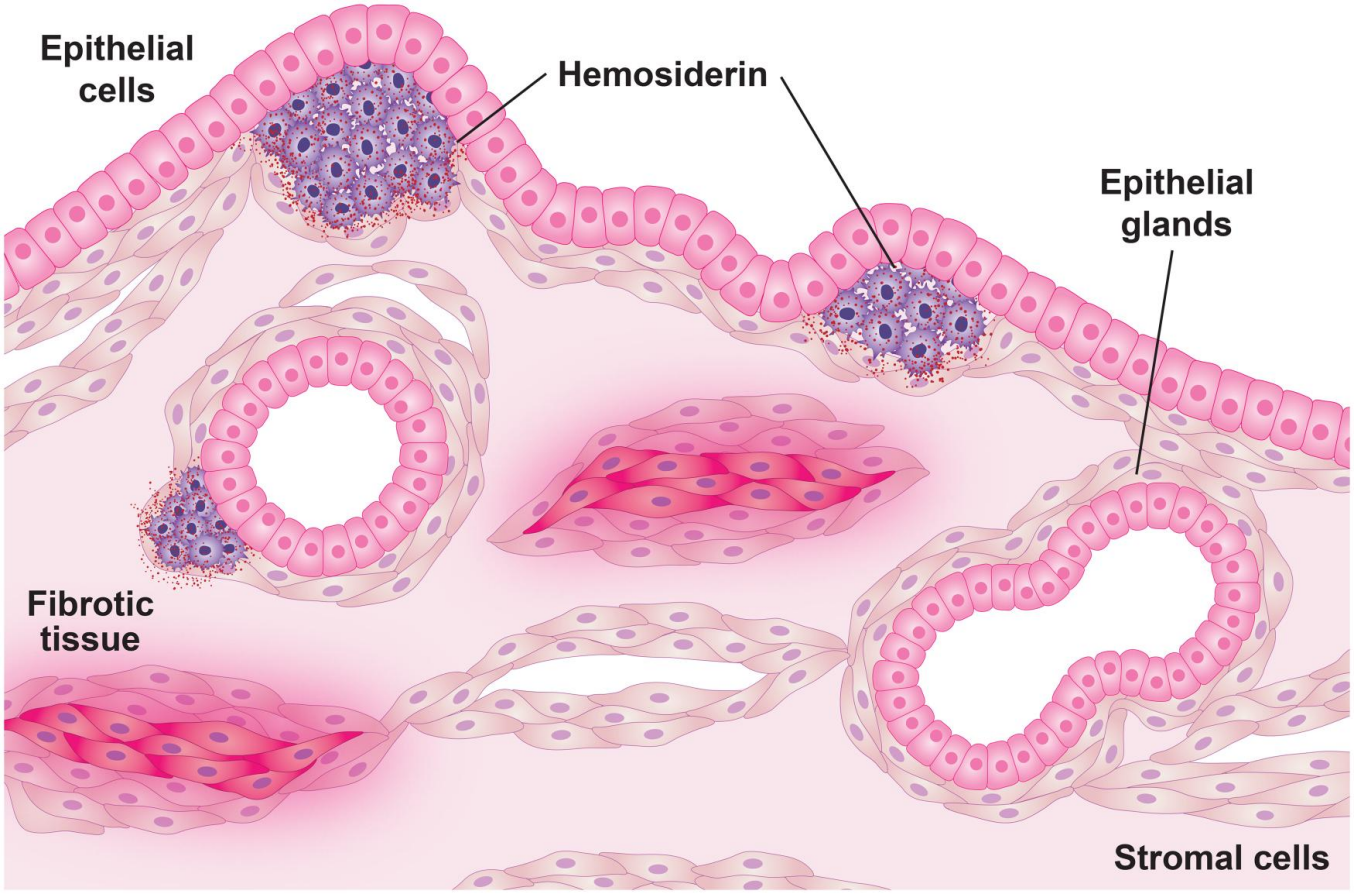
**Schematic of an endometriotic lesion.** Simplified view of an endometriotic lesion sectioned for histological assessment. Lesions characteristically have endometrial epithelial-lined glands, endometrial stroma, hemosiderin-ladened macrophages, and fibrotic tissue.

The consortium defined lesions in rodent models of experimental endometriosis as the establishment of uterine tissue outside the uterine cavity. Acknowledging the caveat that rodent models cannot fully recapitulate human disease, our focus was on the existence of key endometriosis-like lesion characteristics in the models. Endometriosis is defined as immune-infiltrated ectopic endometrial-like tissue, consisting of stromal cells and glandular epithelial cells, the presence of hemosiderin, and an accumulation of ECM consistent with fibrosis (Hsu *et al.*, 2010; Khan *et al.*, 2014; Vigano *et al.*, 2018; Burney, 2022). For the basis of any current or future model, endometriosis-like lesions need to meet these criteria (Fig. 1). We recognize that lesion histology is incredibly diverse and that not all lesions will contain all of the described hallmarks (Clement, 2007; Colgrave *et al.*, 2020). Fibrosis is defined by the pathological accumulation of ECM proteins, resulting in scarring and thickening of the affected tissue (Neary *et al.*, 2015). Historically, fibrosis was not listed as a hallmark of an endometriotic lesion, but the role of tissue remodelling that leads to fibrosis in humans (Sillem *et al.*, 2001; Gilabert-Estelles *et al.*, 2003; Osteen *et al.*, 2003; Gilabert-Estelles *et al.*, 2005; Zhou and Nothnick, 2005; Balkowiec *et al.*, 2018) and animal model lesions (Bruner *et al.*, 1997; Sharpe-Timms *et al.*, 1998; Bruner-Tran *et al.*, 2002; Osteen *et al.*, 2004; Stilley *et al.*, 2010; Stilley and Sharpe-Timms, 2012) is well described in the literature. Thus, our recommendations are updated to reflect the progression of the field to include fibrosis (Vigano *et al.*, 2018). Additionally, lesions from any emerging or future model should be evaluated by an experienced endometriosis researcher and/or a gynaecological pathologist with experience in endometriosis to ensure the resulting histology meets these criteria.

### Mouse model variables

Of the 14 evaluated protocols, key variables were identified, and some were unifiable; however, some could not be unified without jeopardizing future innovation. Key model variables were animal strain, location of ectopic tissue placement, volume of donor tissue, method of induction, hormonal status of recipient, and donor uterine tissue ‘type’. Included are SOPs for pre- and post-operative care of the mice (see Supplementary File S1: EPHect-EM-Homologous-SOP 1 and 10). Below, our recommendations and supporting rationale are described (Table 1).

**Table 1.** Advantages and disadvantages of prototypical homologous mouse models of endometriosis.

	Uterine tissue (full thickness)	Endometrium only	Decidualized ‘menses-like’ endometrium	Mutations/genetics	Deep
No specific skills required for donor tissue preparation	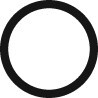	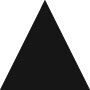	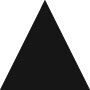	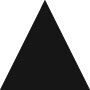	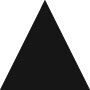
No donor tissue needed	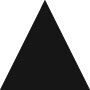	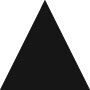	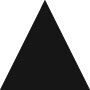	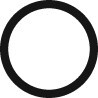	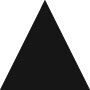
No hormone replacement required in recipient	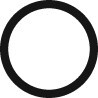	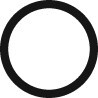	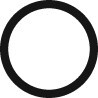	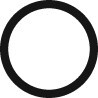	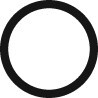
Tissue located in peritoneal cavity (i.e. retrograde menstruation theory)	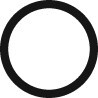	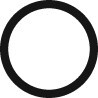	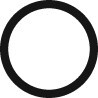	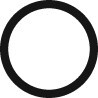	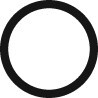
Donor tissue similar to human endometrium	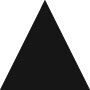 Contains myometrium	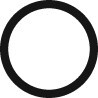	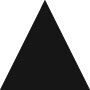 Decidualized tissue dominated by stromal content	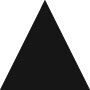 Mutations used are related to both endometriosis and endometrial cancer	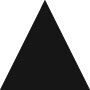 Contains myometrium
Lesions are large, numerous, and easily detectable	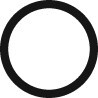	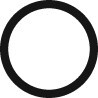	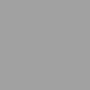	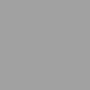	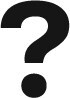
Lesion survival	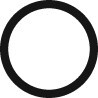 Robust, > 8 months	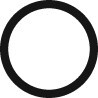 Robust, > 1 year with others noting early resolution	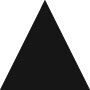 Spontaneous resolution can start within 3 weeks	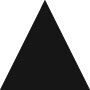 Animals die or need to be euthanized	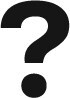
Variation in lesion size mimics human disease	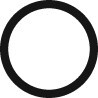	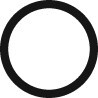	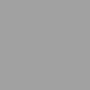	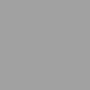	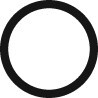 Deep endometriotic
Number of lesions per mouse	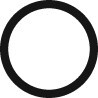 1-3 per animal	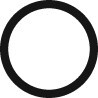 3-6+ per animal	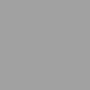 1-4 per animal	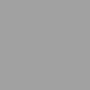 3-6+ per animal	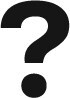
Mature lesions are organized, cystic, stromal and epithelial cell layers, haemosiderin macrophages, and fibrotic tissue	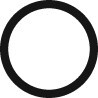 Overabundance of smooth muscle/myometrium compared to human lesions	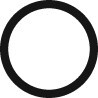	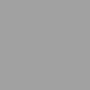 Presence of epithelial glands is highly variable	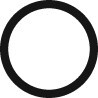	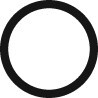 Overabundance of smooth muscle/myometrium compared to human lesions
Lesions are macroscopically and microscopically similar to lesions in human	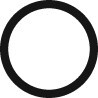	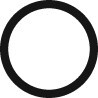	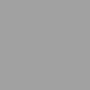 Presence of epithelial glands is highly variable	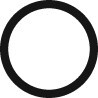	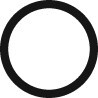
Gene expression similar to human lesions	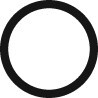	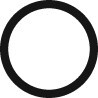	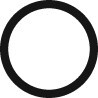	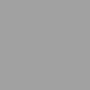 Mutations present uniformly in all endometrial epithelium	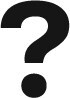
Animals mimic pain symptoms (spontaneous and evoked) similar to women	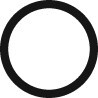	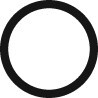	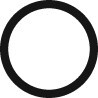	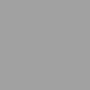 Vaginal bleeding and malignant characteristics may confound results	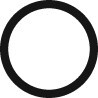
Clinically active drugs effective in reducing endometriosis symptoms	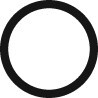	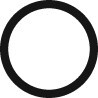	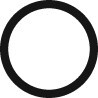	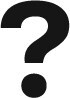	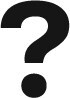
No modification of the peritoneal microenvironment caused by inoculation method	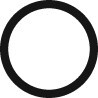	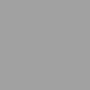 Surgical induction increases innate immune cell infiltration	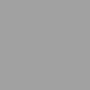 Ovariectomy alters immune dynamics	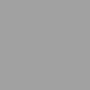 Surgery to develop the model, but no IP injection needed to disperse tissue	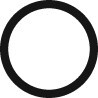 Surgery required to implant the slow-release pumps containing SP and/or CGRP
Use of genetically modified animals	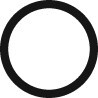	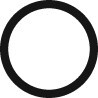	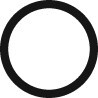	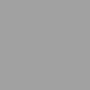 Defined mutations needed for induction	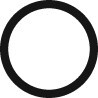 Possible + Substance P and/or CGRP
Lesions are responsive to hormones	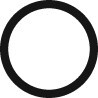	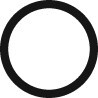	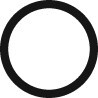	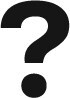	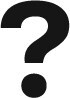
Lesions progress through different stages/types depending on time of necropsy	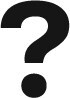	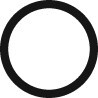	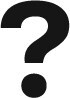	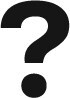	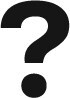
Lesions are easy to identify	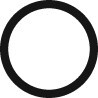	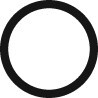	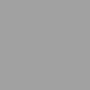	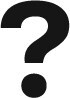	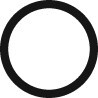
Lineage tracing of specific cell types	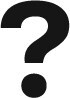	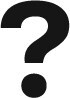	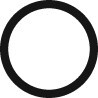	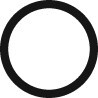 Possible with specific CRE driver	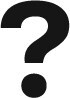
Adverse Side effects	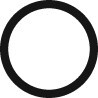	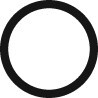	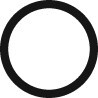	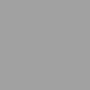 Severe (vaginal bleeding, death); breeding mice with multiple mutations inefficient	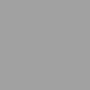 Reduction in body weight after implantation of slow release pumps
Useful to study					
Response to hormones	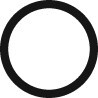	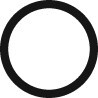	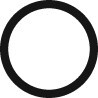	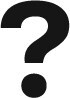	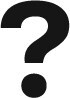
Gene expression changes	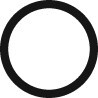	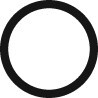	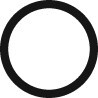	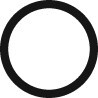 Based on genetic manipulation	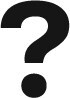
Response to treatment	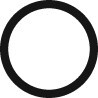	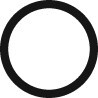	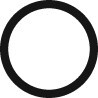	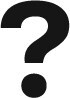	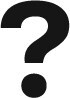
Long-term studies	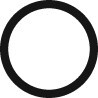	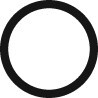	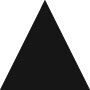	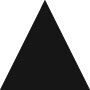	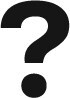
Role of decidualization in endometriosis development	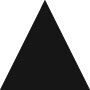	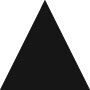	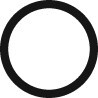	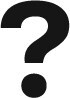	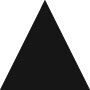
Early implantation of lesions	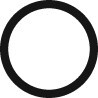	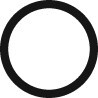	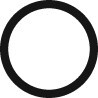	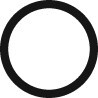	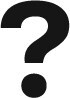
Immune cell changes	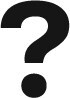	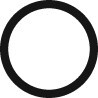	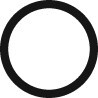	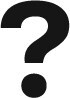	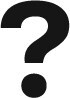

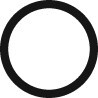
: Yes; this is an advantage of this model.

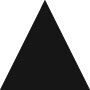
: No; this is a disadvantage of this model.

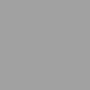
: Maybe; the model is not the best for this.

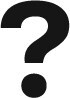
: Unknown; not enough data available to make a claim.

#### Strain

For homologous mouse models, inbred/syngeneic strains are needed to avoid tissue rejection where uterine tissue is transplanted from one mouse (donor) into another (recipient); therefore, the basis for most mouse models include the use of C57BL/6 (Efstathiou *et al.*, 2005; Hirata *et al.*, 2005; Becker *et al.*, 2008; Fainaru *et al.*, 2008; Matsuzaki *et al.*, 2008; Nowak *et al.*, 2008; Altan *et al.*, 2010; Jensen *et al.*, 2010; Körbel *et al.*, 2010; Lu *et al.*, 2010; Pelch *et al.*, 2010; Cheng *et al.*, 2011; Wilkosz *et al.*, 2011; Burns *et al.*, 2012; Pelch *et al.*, 2012; Wieser *et al.*, 2012; Tomio *et al.*, 2013; Cohen *et al.*, 2014; Greaves *et al.*, 2014b; Kim *et al.*, 2014; Kumar *et al.*, 2014; Machado *et al.*, 2014; Pierzchalski *et al.*, 2014; Zhao *et al.*, 2014; Heard *et al.*, 2016; Peterse *et al.*, 2016, 2018a; Ruiz *et al.*, 2016; Dodds *et al.*, 2017; Ferrero *et al.*, 2017; Sanchez *et al.*, 2017; Burns *et al.*, 2018; Jones *et al.*, 2018; Yuan *et al.*, 2018; Chadchan *et al.*, 2019; Forster *et al.*, 2019; Horne *et al.*, 2019; Peyneau *et al.*, 2019; Sekiguchi *et al.*, 2019; Somigliana *et al.*, 1999; Tal *et al.*, 2019; Alali *et al.*, 2020; Fattori *et al.*, 2020, 2024; Hattori *et al.*, 2020; Hayashi *et al.*, 2020; Li *et al.*, 2020; Mishra *et al.*, 2020; Symons *et al.*, 2020; Chen *et al.*, 2021; Ono *et al.*, 2021; Santorelli *et al.*, 2021; Sharma *et al.*, 2021; Dabrosin *et al.*, 2002; Zaninelli *et al.*, 2023) and BALB/c (Bilotas *et al.*, 2010; Chang *et al.*, 2020; Cohen *et al.*, 2015; Dodds *et al.*, 2017; Mattos *et al.*, 2019, Peyneau *et al.*, 2019; Ricci *et al.*, 2011; Ruiz *et al.*, 2016; Sanchez *et al.*, 2017; Uegaki *et al.*, 2015; Woo *et al.*, 2020; Yan *et al.*, 2019a) mice. C57BL/6 mice are more commonly used in endometriosis research as most genetically modified strains are bred on the C57BL/6 background. Other inbred strains used for endometriosis studies, include FVB/N (Dabrosin *et al.*, 2002; Dorning *et al.*, 2021; Jensen *et al.*, 2010), 129S6/SvEv (Becker *et al.*, 2011), and CBA (Peyneau *et al.*, 2019). The use of CD-1, ICR, and ddY (Kusakabe *et al.*, 2010) mice have been described as well; however, these mice are outbred strains that lead to rejection of the uterine tissue, few lesions, and rapid lesion regression (Lee *et al.*, 2009; Umezawa *et al.*, 2009; Kulak *et al.*, 2011; Silveira *et al.*, 2013; Liao *et al.*, 2014; Naqvi *et al.*, 2014; Li *et al.*, 2016; Wilson *et al.*, 2020), unless auto transplantation is performed (Sharpe-Timms, 2002). Of note, the two most common strains of mice used, C57BL/6 and BALB/c, differ in their immunophenotype with C57BL/6 mice being Th1 dominant and BALB/c mice being Th2 dominant (Watanabe *et al.*, 2004). A study by Dodds *et al.* (2017) compared these mouse strains and demonstrated that BALB/c mice had a greater proportion of cystic lesions, while the C57BL/6 mice had a greater variety of lesion types (i.e. cystic, dense, and necrotic). Endometriosis is both immune and hormonally regulated; therefore, immunological differences in the strain of mouse used could lead to diverse conclusions/findings unless these differences are accounted for when designing experiments or interpreting results. Behavioural studies related to animal activity and wellbeing can also differ between strains. For an in-depth discussion of strain-related behavioural responses, see the companion paper by Dodds *et al.* (2025).

#### Location of ectopic tissue placement

In those with endometriosis, lesions are most frequently identified within the peritoneal cavity, with (rare) extra-pelvic lesions found in the skin, pleural cavity, nasal cavity, and some other distant locations (Sasmono *et al.*, 2003; Bagan *et al.*, 2008). Endometriosis is primarily a disease in women and those assigned female at birth; thus, it is modelled in female rodents. Historically, uterine tissue has been grafted both intraperitoneally (Vernon and Wilson, 1985; Sharpe *et al.*, 1990; Sharpe *et al.*, 1991; Pelch *et al.*, 2010; Wilkosz *et al.*, 2011; Burns *et al.*, 2012; Pelch *et al.*, 2012; Greaves *et al.*, 2014b; Mattos *et al.*, 2019; Tal *et al.*, 2019; Chang *et al.*, 2020; Hattori *et al.*, 2020; Li *et al.*, 2020; Symons *et al.*, 2020; Yoshino *et al.*, 2020; Chen *et al.*, 2021; Santorelli *et al.*, 2021), subcutaneously (Cheng *et al.*, 2011; Wang *et al.*, 2013; Wang *et al.*, 2014; Ferrero *et al.*, 2017), and even on the hind leg in studies to evaluate pain mechanisms (Bove, 2016). Subcutaneous engraftment can enable easier measurement of lesion development using callipers for external measurement and facilitating recovery of the lesions at the end of an experiment. This can be particularly beneficial for studies investigating the effects of therapeutics or interventions on lesion size/growth. However, the subcutaneous method does not recapitulate authentic interactions between endometrial and peritoneal tissues and the peritoneal immune microenvironment. Therefore, lesions are most commonly established in the peritoneal cavity of the rodent to allow the uterine tissue to be exposed to the peritoneal niche and sites of attachment most similar to human endometriosis. Placement into the peritoneal cavity, while ideal for most models, can limit measurement (i.e. growth or regression) of lesions depending on location and the depth at which the lesion attaches. Fluorescence and bioluminescence imaging can, however, better enable these endpoints (Burns *et al.*, 2021; Dorning *et al.*, 2021). Peritoneal cavity placement is the most physiologically relevant location for the establishment of endometriotic lesions in mouse models, and the working group was unanimous in their decision to recommend that uterine tissue be placed intraperitoneally (see Supplementary File S1: EPHect-EM-Homologous SOP 5 and 6).

#### Volume of donor tissue

Among the various models, the most commonly used amount of tissue was a donor to recipient ratio of 1:1, which equates to ∼40 mg of non-decidualized uterine tissue containing myometrium (Dodds *et al.*, 2017). Studies that have evaluated different volumes of endometrial tissue have identified that a 1:1 ratio produces the most lesions in the peritoneal cavity (Altan *et al.*, 2010; Burns *et al.*, 2012; Dodds *et al.*, 2017; Forster *et al.*, 2019; Horne *et al.*, 2019; Kim *et al.*, 2020; Woo *et al.*, 2020; Dorning *et al.*, 2021). Studies using a 1:2 ratio of one donor to two recipient mice often report fewer lesions (Somigliana *et al.*, 1999; Hirata *et al.*, 2005; Yoshino *et al.*, 2006; Bacci *et al.*, 2009; Chen *et al.*, 2009; Jensen *et al.*, 2010; Pittaluga *et al.*, 2010; Itoh *et al.*, 2011; Takai *et al.*, 2013; Tomio *et al.*, 2013; Uegaki *et al.*, 2015; Sanchez *et al.*, 2017; Yan *et al.*, 2019a; Fattori *et al.*, 2020; Ono *et al.*, 2021; Fattori *et al.*, 2024; Zaninelli *et al.*, 2023). As the former ratio generates more lesions, for studies focusing on lesion burden as a key endpoint, we recommend using one donor uterus to one recipient mouse. Standardization of tissue volume will improve comparisons across laboratories and treatment outcomes. We also acknowledge that other factors are likely to influence the number of lesions formed (e.g. presence or absence of prior immune challenge in the peritoneal cavity).

#### Method of induction

The most common method of introducing uterine tissue into the peritoneal cavity of mice was via intraperitoneal injection. Initially, in early rodent models of endometriosis, uterine tissue was sutured onto the intestinal mesentery or to the peritoneal wall (Cummings and Metcalf, 1995; Efstathiou *et al.*, 2005; Becker *et al.*, 2008; Fainaru *et al.*, 2008; Matsuzaki *et al.*, 2008; Lee *et al.*, 2009; Umezawa *et al.*, 2009). Importantly, Bain *et al.* (2020) indicated that peritoneal surgery significantly impacts the immune cell profile of the cavity, which may have an impact on lesion attachment and other long-term outcomes. In fact, surgery, especially open abdominal surgery (e.g. laparotomy), can induce stress and impair cell-mediated immunity, accelerating the development of endometriosis (Liu *et al.*, 2016; Long *et al.*, 2016). More recent work has moved away from surgical methods to allow the uterine tissue to seed itself and form unassisted attachments to sites in the peritoneal cavity (Dabrosin *et al.*, 2002; Burns *et al.*, 2012; Li *et al.*, 2016; Jones *et al.*, 2018; Alali *et al.*, 2020; Fattori *et al.*, 2020; Morris *et al.*, 2021). However, this trend may hinder the study of endometriosis lesion interactions attached to specific abdominal organs.

The working group agreed that induction via the intraperitoneal injection method is preferable, and two main methods are used. The first method uses an 18G needle and/or trocar to inject minced uterine tissue into the peritoneal cavity (see Supplementary File S1: EPHect-EM-Homologous SOP 3 and 5) at the midline above the bladder, using 1–2 punctures. This method does not require surgery for the recipient mouse. Mice injected via this method typically have ∼2–3 lesions found near the bladder, adipose tissue, parietal peritoneum, uterus, and intestines, ranging from cystic to dark dense lesions (Hsu *et al.*, 2010; Greaves *et al.*, 2014b; Yuan *et al.*, 2018; Fattori *et al.*, 2020) and do contain the traditional features of endometriotic lesions (Fattori *et al.*, 2020). Alternatively, minced uterine endometrial tissue is injected and dispersed through a small dorsolateral incision (5 mm) that is closed with a clip with no suture to the peritoneal cavity (Burns *et al.*, 2012, 2018; Jones *et al.*, 2018; Morris *et al.*, 2021) (see Supplementary File S1: EPHect-EM-Homologous SOP 3 and 6). In this model, neutrophils and macrophages increase 3–4-fold with the initiation of disease compared to sham-operated animals at 24, 48, and 72 h (Burns *et al.*, 2018). This model develops ∼3–6+ lesions per mouse that begin as white tissue and are red/haemorrhagic by 48–72 h. By 2 weeks, lesions are cystic and exhibit organized glands, stroma, and haemosiderin macrophages, and are fibrotic (Burns *et al.*, 2018). These lesions are found attached to ovarian adipose tissue, intestines, cul-de-sac, peritoneal wall, stomach, diaphragm, uterus, and bladder and are maintained for up to a year (experimental testing was stopped at 1 year) in this mouse model (Jones *et al.*, 2018). Alternatively, one research group described endometriosis induction via laparoscopy (Peterse *et al.*, 2016, 2018b, 2024). The advantage of laparoscopically guided inoculation is that uterine tissue can be placed at locations where lesions are most often removed in women, namely the peritoneal wall and cul-de-sac. Using this model, up to 60% of the implanted uterine tissue pieces could be retrieved after 1 week (Peterse *et al.*, 2016, 2018a, 2024). The main disadvantage of this model is that it requires specialized equipment and a highly skilled researcher to perform the laparoscopy (Corona *et al.*, 2011).

#### Hormonal status

Endometriosis is a hormonally responsive disease in women, where endometriotic lesions are exposed to cyclical fluctuations of reproductive hormones throughout the menstrual cycle. A large number of mouse studies use exogenous hormones (oestrogen alone, oestrogen followed by progesterone, or pregnant mare serum gonadotropin (PMSG) to synchronize the donor uterine tissue prior to being used to initiate endometriosis (Dabrosin *et al.*, 2002; Hirata *et al.*, 2005; Yoshino *et al.*, 2006; Bacci *et al.*, 2009; Altan *et al.*, 2010; Jensen *et al.*, 2010; Pittaluga *et al.*, 2010; Itoh *et al.*, 2011; Burns *et al.*, 2012; Wieser *et al.*, 2012; Takai *et al.*, 2013; Tomio *et al.*, 2013; Greaves *et al.*, 2014b; Pierzchalski *et al.*, 2014; Kim *et al.*, 2014, 2020; Liao *et al.*, 2014; Ruiz *et al.*, 2016; Sanchez *et al.*, 2017; Burns *et al.*, 2018; Jones *et al.*, 2018; Peterse *et al.*, 2018a; Yuan *et al.*, 2018; Forster *et al.*, 2019; Horne *et al.*, 2019; Sekiguchi *et al.*, 2019; Somigliana *et al.*, 1999; Yan *et al.*, 2019a; Alali *et al.*, 2020; Fattori *et al.*, 2020; Li *et al.*, 2016, 2020; Dorning *et al.*, 2021; Ono *et al.*, 2021; Fattori *et al.*, 2024; Zaninelli *et al.*, 2023) (see Supplementary File S1: EPHect-EM-Homologous SOP 2, 3, and 4). Other studies have taken donor uterine tissue in oestrus (Nowak *et al.*, 2008; Wang *et al.*, 2013; Dodds *et al.*, 2017); however, this method requires many donor animals on hand to ensure oestrus on the day of surgery. Due to these differences in study design, our recommendation is that investigators be consistent in their studies to use hormone-supplemented donors or to use cycle-staged donors. For the recipient, we recommend that experimental endometriosis is established in mice with intact ovaries to mimic the cyclical hormonal fluctuations that naturally occur. Early mouse models of endometriosis often utilized ovariectomy (see Supplementary File S1: EPHect-EM-Homologous SOP 9) with supplementation of oestrogen to reduce hormonal variation. Studies have found that exogenous oestrogen increases lesion size but not lesion number (Burns *et al.*, 2012; Burns *et al.*, 2018; Jones *et al.*, 2018; Morris *et al.*, 2021). Nevertheless, using intact mice has become increasingly common to more closely mimic human disease and avoid surgery. For studies evaluating hormones or hormonal regulation, ovariectomy followed by hormonal supplementation may be required as part of the experimental design, although natural cycling is required when testing potential therapeutics to determine their impact on cyclicity. When using intact animals, Dodds *et al.* (2017) found a slight difference in the number of lesions that attached when tissue was injected during oestrus (relatively lower oestradiol levels) versus the proestrus phase (relatively higher oestradiol levels) of the cycle; metestrus and dioestrus were not examined. In contrast, other investigators have not observed any correlation between lesion number and cycle stage at tissue injection ((Dorning *et al.*, 2021) and unpublished data). While the cycle stage at induction may have minor impacts on tissue attachment, the cycle stage at necropsy is more important to control as hormone status (see Supplementary File S1: EPHect-EM-Homologous SOP 8) impacts expression of genes and proteins in endometriosis lesions (i.e. progesterone receptor (PGR), matrix metalloproteinases (MMPs), lactoferrin (LTF), etc.) (Burns *et al.*, 2012). With the advent of static isolated cages, mice are no longer exposed to male pheromones, and consequently do not cycle as regularly; therefore, we recommend that male bedding be placed in the female cages every other day to every third day to expose the female mice to pheromones secreted by the male mice (Whitten *et al.*, 1968; McCarthy *et al.*, 2018).

#### Donor uterine tissue ‘type’

To allow for innovation of experimental endometriosis models in the future, we cannot recommend standardization of the donor uterine tissue ‘type’. However, it is appropriate to discuss different types of tissue currently being used to induce experimental endometriosis. The aetiology of endometriosis remains unknown, but most models begin with uterine tissue with/without myometrium, with the aim of recapitulating retrograde menstruation or at least to establish ectopic endometrial-like tissue engraftment. In support of this approach, other tissue types (e.g. lung, mammary gland, and bladder) minced and injected into the peritoneal cavity do not attach or form endometriosis-like lesions; however, other investigators show that adipose tissue does attach and can attract blood vessels and nerve fibres but does not develop the typical endometriotic features of glands or stroma (Castro *et al.*, 2021; Peterse *et al.*, 2024). Importantly, menstrual effluent from women consists of epithelial cells, decidualized stromal cells, and multiple immune cell types (Salamonsen and Lathbury, 2000; Salamonsen, 2021). To represent these cell types likely involved in endometriosis development, three donor uterine tissue ‘types’ have emerged for use in the mouse model, all with their own pros and cons.

##### Full-thickness uterus incorporating myometrium

The use of full-thickness uterine tissue produces endometriosis-like lesions that are often cystic. These lesions have epithelial-lined glands, stroma, and hemosiderin-laden macrophages and exhibit fibrosis (Lu *et al.*, 2010; Peterse *et al.*, 2016; Ferrero *et al.*, 2017; Yan *et al.*, 2019a; Fattori *et al.*, 2020). Importantly, lesions remain viable and robust in the mouse for long-term studies (Dorning *et al.*, 2021). An advantage to this model is that specific skills are not required to prepare the donor tissue or to inject minced uterine tissue, making the full-thickness model an attractive model for ease and higher throughput of animals. Lesions derived from full-thickness uterine tissue frequently contain the key hallmarks of lesions, are larger, and exhibit a greater bioluminescent signal in a model implemented for non-invasive monitoring of lesions. Compared to a model utilizing decidualized ‘menses-like’ endometrium, ∼35% more lesions were evident 6 weeks after model induction (Dorning *et al.*, 2021); thus, the full-thickness model exhibits significantly less spontaneous resolution of lesions. Functionally, the lesions are hormone- and treatment-responsive in growth and gene expression changes. Mice exhibit an increase in both spontaneous and evoked pain-like behaviours compared to sham animals (Lu *et al.*, 2010; Fattori *et al.*, 2020). A weakness of the model is that the injection of full-thickness uterine tissues results in the incorporation of myometrial tissue into lesions. While endometriosis lesions in women often show staining for smooth muscle actin (Anaf *et al.*, 2000; Barcena de Arellano *et al.*, 2011) and the presence of cells with a myofibroblast phenotype, it is unlikely that the tissue type injected and the amount of muscle tissue present in this model fully mimic human disease. As with all models, the amount of tissue injected into the peritoneal cavity does not correspond to the number of lesions formed. The lesions are variably located, of variable sizes, and can be difficult to locate and quantify if fluorescence/bioluminescence is not used.

The preparation of the uterine tissue for implantation in this model is straightforward, easy, and fast, which allows for inducing large numbers of animals for generating dose–response curves to test the efficacy of potential drugs. The lesions produced in this model have been induced by either injecting minced uterine tissue from donor animals into the peritoneal cavity of genetically identical recipient mice using an 18G needle (see Supplementary File S1: EPHect-EM-Homologous SOP 5) (Fattori *et al.*, 2020) or by performing laparotomy and suturing uterine tissue onto the bowel mesentery, similar to what has been described in Supplementary File S1: EPHect-EM-Homologous SOP 12 (Bilotas *et al.*, 2010; Pelch *et al.*, 2010), although the latter has become obsolete in mice in recent years. Endometriosis mouse models induced with this type of tissue have been used to study early development of endometriosis lesions, angiogenesis, endometriosis-associated pain, and endometriosis-associated infertility, and to identify new therapeutic treatment options or to examine the efficacy of re-purposed FDA-approved drugs (Lin *et al.*, 2006; Bilotas *et al.*, 2010; Lu *et al.*, 2010; Ruiz *et al.*, 2016; Fattori *et al.*, 2020; Elsherbini *et al.*, 2022; Maddern *et al.*, 2022) for endometriosis. In addition, investigators have used this model to optimize a laparoscopic procedure for endometriosis induction as well as to investigate immune changes in the peritoneum in response to lesion development (Peterse *et al.*, 2016; He *et al.*, 2022). Other investigators have used this model to study neuroimmune communication in endometriosis. They showed that in mice, nociceptor ablation reduced pain, monocyte recruitment, and lesion size (Fattori *et al.*, 2024). In general, this model is very versatile and can be used to study pathogenesis as well as the pathophysiology of endometriosis.

##### Endometrium/uterus without myometrium

Similar to the full-thickness model, obtaining endometrial tissue by peeling away the myometrium in layers from the outside of the uterus (as opposed to scraping the endometrium away from the myometrium) or using sharp dissection to isolate the endometrial layer (Dodds *et al.*, 2017; Dorning *et al.*, 2021) produces lesions that are often cystic, dense, and/or haemorrhagic at different stages post-inoculation (Burns *et al.*, 2018). Lesions are typically found attached to uterine arteries, cul-de-sac, fat pads or peritoneal wall, or near the spleen/stomach area and intestine; however, this can be somewhat dependent on the angle of tissue injection. Lesions are typically not found on the liver, kidney, or ovary. These lesions have epithelial-lined glands, stroma, fibrosis, and hemosiderin, but have less myometrium than the full-thickness model (Burns *et al.*, 2012; Dodds *et al.*, 2017; Burns *et al.*, 2018; Jones *et al.*, 2018; Dorning *et al.*, 2021; Morris *et al.*, 2021). Lesions derived from the endometrium model survive a minimum of 2 months. Lesion number, size, and appearance are variable, based on the method used to dissect the endometrium before implantation. Functionally, lesion growth and gene expression changes are hormonally responsive (Burns *et al.*, 2012, 2018; Jones *et al.*, 2018). Animals in this model exhibit pain-like symptoms, with a reduction in abdominal reaction threshold in response to von Frey filaments (mechanical hyperalgesia), but no differences in paw withdrawal was observed compared to sham-operated animals (Dorning *et al.*, 2021). As with all injection models, one weakness is that the amount of tissue inoculated into the peritoneal cavity does not necessarily correspond to the number of lesions formed. In addition, the lesions are variably located, are of variable sizes, and can be difficult to locate if fluorescence/bioluminescence is not used.

The use of endometrial tissue for the development of endometriosis lesions recapitulates the appearance of endometriotic lesions with the presence of epithelial cells, glands, and stroma. The lesions from this model are induced by injecting homogenized endometrial tissue by needle (18G) or surgically injecting minced endometrium into the peritoneal cavity of the mouse (Burns *et al.*, 2012; Dodds *et al.*, 2017; Burns *et al.*, 2018; Jones *et al.*, 2018; Dorning *et al.*, 2021; Morris *et al.*, 2021) (see Supplementary File S1: EPHect-EM-Homologous SOP 3, 5, or 6). While this model requires trained individuals to prepare the donor endometrium, this version of the model offers several benefits; lesions are found in the peritoneal cavity, are larger or more numerous than some models, survive in the peritoneal cavity long-term, develop into mature lesions, and are varied in size and location. Lesions found in the peritoneum range from dense to cystic lesions that are responsive to hormones, therapeutics, and/or endocrine-disrupting compounds (Burns *et al.*, 2012; Dodds *et al.*, 2017; Burns *et al.*, 2018; Jones *et al.*, 2018; Dorning *et al.*, 2021; Morris *et al.*, 2021). This model can and has been used to study early implantation of lesions, immune-mediated changes in response to lesion development, lesion survival, and changes in pain sensory behaviour.

##### ‘Menses’-like decidualized endometrium

In humans, the functional layer of the endometrium, including endometrial epithelial and stromal cells, undergoes spontaneous decidualization and is shed during menstruation. In contrast, rodents have an oestrous cycle, the endometrium does not spontaneously decidualize (Ng *et al.*, 2020), and the endometrium is not shed but is instead remodelled during the cycle. To recapitulate menstruation, donor mice undergo a truncated human hormonal schedule, artificial decidualization is induced by injecting sesame or corn oil into a uterine horn, and shedding is initiated by progesterone withdrawal (Finn and Pope, 1984; Cousins *et al.*, 2014; Greaves *et al.*, 2014b; Peterse *et al.*, 2018b; Horne *et al.*, 2019; Forster *et al.*, 2019; Kim *et al.*, 2020) (see Supplementary File S1: EPHect-EM-Homologous-SOP 4). The decidualized donor tissue is used to establish lesions in recipient mice with the aim of mimicking retrograde menstruation. In contrast to human decidualized tissue, which does maintain glandular structures, a drawback to the decidualized rodent endometrium is that it contains mostly decidualized stromal cells, with few epithelial glands (Peterse *et al.*, 2018b, 2024). Moreover, variation has been observed in the degree of decidualization and the amount of decidualized endometrium in donor mice. In mice, during decidualization, there are often areas of the endometrium that lack decidualization; thus, these non-decidualized areas still contain the normal endometrial glandular structures (Finn and Pope, 1984; Brasted *et al.*, 2003; Xu *et al.*, 2007; Rudolph *et al.*, 2012; Peterse *et al.*, 2018b, 2024).

In recipient mice, endometriosis lesions in this model are easy to identify as they are often red at 2–3 weeks post induction. The lesions are found attached to the peritoneal wall, fat, bladder and, occasionally, the intestines. Not all the injected tissue attaches, and floating material can be identified during laparotomy for lesion recovery (Greaves *et al.*, 2014b; Dorning *et al.*, 2021). Some lesions in this model exhibit spontaneous regression from 3 weeks post tissue inoculation and do not contain as many epithelial gland structures as models that use naïve endometrium or full-thickness uterine tissue (Dorning *et al.*, 2021). Other investigators indicated that endometriosis lesions in this model rarely contained epithelial glands but do often contain hemosiderin-laden macrophages (Peterse *et al.*, 2018a, 2024). Levels of fibrosis are comparable to other models (Dorning *et al.*, 2021), indicating this is a consistent feature of lesions recovered from other experimental endometriosis models.

The use of decidualized stroma for the development of endometriosis lesions in mice represents the development of endometriosis lesions from endometrium, including decidualized stroma, in humans. The differentiation process of decidualization does not happen spontaneously in mice; thus, decidualized tissue is acquired through hormonal and mechanical processes. The use of ‘menses-like’ endometrium in the peritoneal cavity to develop endometriosis lesions can be done with or without hormone replacement in the recipient and allows for early implantation studies and studies investigating the contribution of the immune system (Forster *et al.*, 2019; Cousins *et al.*, 2020; Hogg *et al.*, 2020, 2021; Dorning *et al.*, 2021; Greaves *et al.*, 2014a,b, 2015, 2017b). Using this model, mice exhibit pain-like behaviours and molecular alterations in the peripheral and central nervous system 2–3 weeks post lesion induction that mimic human disease and associated maladaptation in the nervous system (Greaves *et al.*, 2017b). Lesions are infiltrated by abundant macrophages that drive lesion pathogenesis (Hogg *et al.*, 2020) and are associated with the generation of pain-like behaviours (Forster *et al.*, 2019). Macrophage infiltration as well as neuroangiogenesis are regulated by oestrogen signalling in this model (Greaves *et al.*, 2014a, 2015). Moreover, Greaves *et al.* have used this model to identify a protective population of monocyte-derived peritoneal macrophages that, when present in abundance, result in fewer and smaller lesions (Hogg *et al.*, 2021). This supports the theory that women with endometriosis exhibit immune dysfunction and that aberrations in macrophage function are linked with lesion persistence. This preclinical model has therefore also been used to test the repurposing potential of drugs that target metabolic processes as a non-hormonal treatment for endometriosis (Horne *et al.*, 2019).

### Rat model variables

From the evaluated protocols and publications on the rat model of endometriosis, far fewer differences were found among the protocols than were identified in publications using mice. Key variables for the rat model were strain, location of ectopic tissue placement, volume of tissue, and hormonal status. Methods of induction and tissue type were mostly similar in all protocols. Below, we describe our recommendations and their rationale (Table 2).

**Table 2.** Advantages and disadvantages in prototypical homologous rat models of endometriosis.

	Uterine tissue (full thickness)	Decidualized ‘menses-like’ endometrium IP injection
No specific skills required for donor tissue preparation	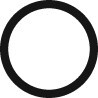	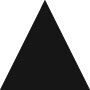
No donor tissue needed	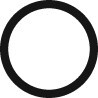	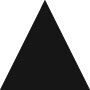
No hormone replacement required	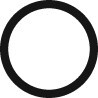	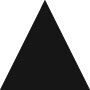
Mimics retrograde menstruation theory	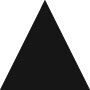	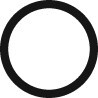
Donor tissue similar to human endometrium	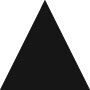 Contains myometrium	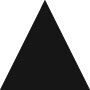 Decidualized tissue dominated by stromal content
Lesions are large, numerous, and easily detectable	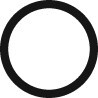	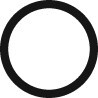 When lesions are present
Lesion survival	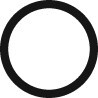 + 2 months	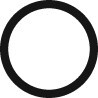 + 3 months when present
Variation in lesion size mimics human disease	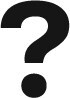	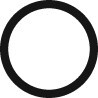 When lesions are present
Number of lesions per rat	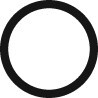 4 sutured per animal	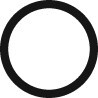 1-4 per animal when present
Mature lesions are organized, cystic, stromal and epithelial cell layers, haemosiderin, macrophages, and fibrotic tissue	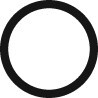 Overabundance of smooth muscle/myometrium compared to human lesions	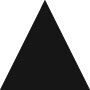 Epithelial glands are not common
Lesions are macroscopically and microscopically similar to lesions in women	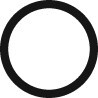	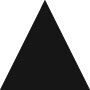 Epithelial glands are not common
Gene expression similar to human lesions	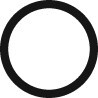	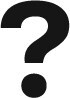
Animals mimic pain symptoms (spontaneous and evoked) as found in women	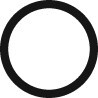	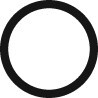 Spontaneous ongoing pain symptoms
Clinically active drugs are effective in reducing endometriosis symptoms in rats	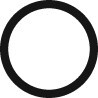	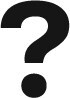
IP injection causes little to no modification of the peritoneal microenvironment	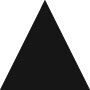 Uterine tissue is sutured into intestinal mesentery	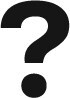
Use of genetically modified animals	Possible	Possible
Lesions are responsive to hormones	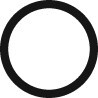	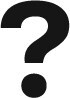
Lesions progress through different stages/types depending on time of necropsy	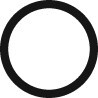	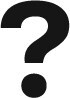
Lesions are easy to identify	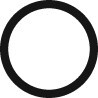	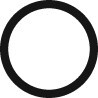
Lineage tracing of specific cell types	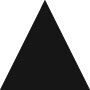	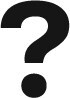
Adverse side effects	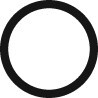	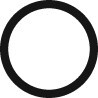
Useful to study		
Response to hormones	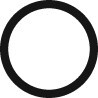	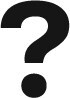
Gene expression changes	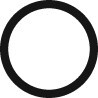	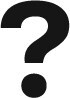
Response to treatment	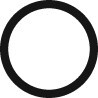	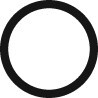
Long-term studies	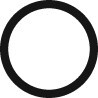	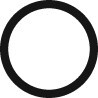
Role of decidualization in endometriosis development	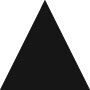	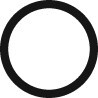
Early implantation of lesions	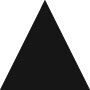	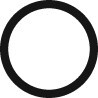
Immune cell changes	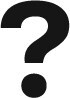	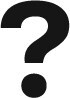

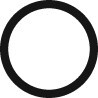
: Yes; this is an advantage of this model.

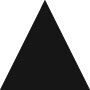
: No; this is a disadvantage of this model.

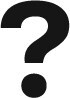
: Unknown; not enough data available to make a claim.

#### Strain

Most studies in rat, to date, have used the outbred albino Sprague-Dawley strain, likely due to their docility, ease of handling, regular reproductive cyclicity, and the ability to develop transgenic models (Sharpe-Timms, 2002). Far fewer studies have used the Wistar rat (also outbred), which tend to be slightly smaller in size.

#### Location of ectopic tissue placement

An autologous rat (Sprague-Dawley) surgical model was originally developed in 1985 (Vernon and Wilson, 1985). Four full-thickness uterine squares from the distal third of one uterine horn were auto-transplanted onto the arterial cascades of the small intestine (see Supplementary File S1: EPHect-EM-Homologous SOP 11, 12, and 15). The implant size increased over time, reaching maximal growth at 60 days post transplantation (Vernon and Wilson, 1985). The endometriosis implants developed into cyst-like structures and, depending on the stage of the oestrous cycle or hormonal treatments, could be fluid-filled. Initial studies compared the growth of the implants to sham-controls and to the injection of uterine lavage or endometrial scrapings into the peritoneal cavity but found that the only viable endometriosis implants were those from the surgical implantation procedure (Vernon and Wilson, 1985). With this knowledge, more recent studies compared endometrial tissue implants to fat implants or suture ties (Sharpe-Timms, 2002).

Other variations of rat models for endometriosis include subcutaneous placement of endometrial tissue fragments. An initial study placed endometrial tissue sections subcutaneously in Sprague-Dawley rats to examine implant regression after ovariectomy and during pregnancy (Vernon and Wilson, 1985). A second study implanted tissues onto the muscle of the inguinal region in Wistar rats to demonstrate the effects of drugs on reducing implant size (Pereira *et al.*, 2015). In a third study, subcutaneous endometrial tissue fragments in Wistar rats were used to evaluate the effect of oestrogenic properties of avocado seed extract on macroscopic implant regression (Minko Essono *et al.*, 2020). However, to account for consistency in lesion size, distal proximity to the utero-ovarian vasculature, and development of the lesions produced, the placement of fragments on the arterial cascades is the recommended option.

#### Volume of tissue

Studies using the auto-transplantation model modified the number of implants from four to six full-thickness uterine squares and initiated studies at 4 weeks post auto-transplantation (Vernon and Wilson, 1985; Sharpe *et al.*, 1990; Sharpe *et al.*, 1991; Wright and Sharpe-Timms, 1995; Sharpe-Timms *et al.*, 1998; Cox *et al.*, 2001; Sharpe-Timms, 2002; Stilley *et al.*, 2009; Stilley and Sharpe-Timms, 2012; Birt *et al.*, 2013b; Sharpe-Timms *et al.*, 2020). Whether using four or six implants, the lesions remain viable for approximately 1 year in duration if initially established in young adult animals.

Despite the surgical nature of this model, studies of developing endometriotic implants in rat uterine auto-transplantation models aided the discovery of mechanisms of the genesis of endometriotic implants. For example, endometriotic implants at 36 h, 2 weeks, and 4 weeks post-surgery progressed from pale avascularized implants to red vascularized lesions, to well-established encapsulated cysts that express enzymes, playing a role in peritoneal remodelling and progression of the implants. Others have also observed angiogenesis as well as neurogenesis developing as early as 7 days after surgery (Torres-Reveron *et al.*, 2018a).

#### Hormonal status

As with the mouse model, the growth of endometrial tissue is oestrogen-dependent; thus, the recommended hormonal status of the rat is to be hormonally intact unless the research goal is to study hormones or hormonal regulation. Like humans, regression of uterine ectopic implants can be induced in rats by generating a hypo-oestrogenic state, either by ovariectomy, application of GnRH agonists or synthetic compounds (Sharpe *et al.*, 1990; Sharpe *et al.*, 1991; Wright and Sharpe-Timms, 1995; Sharpe-Timms *et al.*, 1998; Yavuz *et al.*, 2007; Yuan *et al.*, 2012). Proper cycling must be ascertained before endometriosis induction and should be monitored at various points during the experimental protocol if therapeutic interventions are being tested to account for impact on cyclicity (whether on alterations in length of cycle or proportion of time spent in each stage). Like mouse models, the oestrous cycle stage at necropsy must be determined if examining hormonally responsive changes (see Supplementary File S1: EPHect-EM-Homologous SOP 14). If ovariectomized rats are used, supplementing with oestradiol is recommended for lesion growth.

The rat model has been useful for studies of pain, fertility, stress, hormone modulation, ovarian function, immunomodulation, inflammation, gene expression and epigenetic regulation, protein synthesis and secretion, therapeutic modulation of the implants, and others (Vernon and Wilson, 1985; Rajkumar *et al.*, 1990; Barragan *et al.*, 1992; Moon *et al.*, 1993; Sharpe and Vernon, 1993; Nothnick *et al.*, 1994; Sharpe-Timms *et al.*, 1998; Sharpe-Timms, 2002; Uchiide *et al.*, 2002; Cason *et al.*, 2003; Berkley *et al.*, 2004; Rojas-Cartagena *et al.*, 2005; Berkley *et al.*, 2007; Flores *et al.*, 2007; Zhang *et al.*, 2008a; McAllister *et al.*, 2009; Stilley *et al.*, 2009, 2010; Cuevas *et al.*, 2012; Birt *et al.*, 2013a,b; Appleyard *et al.*, 2015, 2020, 2021; Hernandez *et al.*, 2015, 2017; Torres-Reveron *et al.*, 2016, 2018a,b, 2020; Cuevas *et al.*, 2018; Lopez *et al.*, 2020; Seguinot-Tarafa *et al.*, 2020). The rat model of endometriosis mirrors many end-points of the disease in patients, including ectopic lesions, impaired natural killer cell activity, protein synthesis, and secretion by the ectopic lesions (Sharpe and Vernon, 1993; Sharpe *et al.*, 1993), increased inflammatory mediators (in peritoneal fluid, serum, lesions, and endometrium), elevation of angiogenic molecules, upregulation of nerve growth factors and their receptors, damage to the intestinal tract, increased vaginal hyperalgesia, and changes in the central pathways of pain modulation (Vernon and Wilson, 1985; Moon *et al.*, 1993; Cason *et al.*, 2003; Berkley *et al.*, 2004, 2007; Grummer, 2006; Zhang *et al.*, 2008b; McAllister *et al.*, 2009; Cuevas *et al.*, 2018). Endometriotic implants in rats are histologically alike, respond to steroids, and activate similar inflammatory mechanisms to those that characterize human disease, with a comparable global gene expression profile (Nothnick *et al.*, 1994; Sharpe-Timms, 2002; Uchiide *et al.*, 2002; Rojas-Cartagena *et al.*, 2005; Flores *et al.*, 2007; Seguinot-Tarafa *et al.*, 2020). Compared to the mouse, the rat appears better suited to investigations on fertility and those using complex behavioural studies. The rat model is well suited to evaluations incorporating aspects of gastrointestinal physiology and cross-organ sensitization due to the lesions being sutured on to the intestinal mesentery (Torres-Reveron *et al.*, 2016; Lopez *et al.*, 2020; Torres-Reveron *et al.*, 2018a, 2020). The contribution of stress to the development and progression of endometriosis is also observed in the rat model (Cuevas *et al.*, 2012; Appleyard *et al.*, 2015, 2020; Hernandez *et al.*, 2017), as well as the positive contribution of environmental enrichment and exercise (Hernandez *et al.*, 2015; Torres-Reveron *et al.*, 2018b; Appleyard *et al.*, 2021). An important attribute to using a rat model is that many pharmacokinetic studies are initially done in rats. For drug discovery work, traditional adsorption, distribution, metabolism and excretion, work is often done in rats to examine toxicity and pharmacokinetics of the compound.

### Emerging models

#### Cancer driver mutations

Somatic cancer driver mutations are observed across all subtypes of endometriosis (Anglesio *et al.*, 2017; Chui *et al.*, 2017; Guo, 2018a; Suda *et al.*, 2018, 2019; Bulun *et al.*, 2019; Lac *et al.*, 2019b; Praetorius *et al.*, 2022; Orr *et al.*, 2023). While significant differences between subtypes have not been described, smaller cohorts suggest that ovarian endometriomas have greater mutation burdens and increased levels of mutational heterogeneity (Praetorius *et al.*, 2022). Mutational analysis shows that recurrent cancer driver mutations are found exclusively in the epithelial compartment of endometriotic lesions and normal-appearing endometrial tissue (Lac *et al.*, 2019a; Moore *et al.*, 2020; Yamaguchi *et al.*, 2022). In support, somatic non-driver mutations observed in the stromal compartment of lesions are less frequent and do not appear to be recurrent (Lac and Huntsman, 2018; Noe *et al.*, 2018; Suda *et al.*, 2019). The invasive phenotype of endometriosis shares aspects of tumour metastasis and invasiveness, including migration, anchorage-independent growth, recruitment of vasculatures, and survival at distal sites (Giudice and Kao, 2004; Cheng *et al.*, 2011; Wilson *et al.*, 2020). Cancer driver mutations are thought to have invasive cell properties and to promote lesion establishment and growth. For example, patients with more severe stages of endometriosis are more likely to have lesions that contain cancer driver mutations (Praetorius *et al.*, 2022; Orr *et al.*, 2023). Importantly, somatic mutations are not exclusively a marker of malignant potential.

Mutant mouse models carrying cancer driver mutations support a role for these mutations in the establishment and spread of invasive endometrial-like tissue (Dinulescu *et al.*, 2005; Cheng *et al.*, 2011; Wilson *et al.*, 2020). Given the natural tendency of cancer driver mutations to generate invasive cell properties, which could have selective advantages for endometrial tissue in the peritoneal cavity, lesion establishment may not be as dependent on endometrial tissue transplantation (Wilson *et al.*, 2020) or the use of exogenous hormones (Cheng *et al.*, 2011) in these mouse models. Cancer driver mutations may promote additional aspects of disease pathogenesis, such as changes to the lesion microenvironment and hormone responsiveness. The existence of genetically engineered mouse or rat strains to study cancer-related processes *in vivo* offers the opportunity to study the effects of somatic mutations and other genetically traceable alterations on pathogenic mechanisms in endometriosis rodent models (Dinulescu *et al.*, 2005). Additional improvements to existing models or the establishment of new models may allow for time-based control of mutation induction or the ability to study cancer driver mutations specifically in ectopic lesions, avoiding the unnecessary phenotypic effects of mutations in eutopic endometrium. For example, early evidence for the role of cancer-associated mutations in endometriosis came from the inadvertent induction of endometriosis-like lesions in an ovarian cancer mouse model, by injecting a cre-expressing virus into the LSL-KRAS(G12D) transgenic mouse strain (Dinulescu *et al.*, 2005). Transplant models offer the ability to study cancer driver mutations in ectopic lesions, avoiding the confounding phenotypic effects of mutations in eutopic endometrium. On the other hand, somatic cancer driver mutations are not only observed in endometriosis lesions but have also been found in histopathologically normal-appearing endometrium of women with and without endometriosis. It is currently unknown whether cancer driver mutations in eutopic endometrium are causative of endometriosis development, thus, co-modelling of ectopic and eutopic genetically modified endometrium may be desirable.

#### Deep endometriosis

One subtype of endometriosis that often causes severe pain in those with endometriosis and poses a challenge in clinical management is deep endometriosis (Tosti *et al.*, 2015). While a baboon deep endometriosis model was established a decade ago, the model requires facilities and expertise for the use of non-human primates (Donnez *et al.*, 2013). This model is cost-prohibitive and may have similar experimental limitations as humans; hence, the use of this model is not feasible for most laboratories. Capitalizing on the finding that neuropeptides, such as substance P and calcitonin gene-related peptide (CGRP), secreted by sensory nerves surrounding the endometriotic lesions actively facilitate lesion progression and fibrogenesis (Liu *et al.*, 2019; Yan *et al.*, 2019b), a deep endometriosis mouse model can be established by chronic infusion of substance P and/or CGRP in a conventional endometriosis mouse model. This model differs from the conventional rodent models of endometriosis by fulfilment of the four basic requirements for deep endometriotic lesions: (i) the resultant lesions consist of endometrial stromal and epithelial cells, (ii) lesions are encapsulated in surrounding tissues or organs (e.g. bowel wall, rectovaginal septum), (iii) lesions display signs consistent with smooth muscle metaplasia, and (iv) lesions exhibit extensive fibrosis (Yan *et al.*, 2019a). As such, this model offers a viable and much more economical option than the baboon deep endometriosis model. In addition, this model is based on known biological mechanisms underlying the deep endometriosis formation. It also provides an experimental explanation for a key difference between deep endometriosis and ovarian endometrioma: deep endometriosis lesions in humans are always located, without exception, in proximity to various nerve plexuses in the pelvic cavity (Foti *et al.*, 2018).

#### Endometrial injection model in rats

In recent years, rat injection models have begun to be developed in a similar manner to the mouse injection model. Persoons *et al.* (2020) induced endometriosis in adult Sprague-Dawley rats, adapting a protocol based on the menstruating mouse model. In brief, menstrual endometrial tissue separated from the myometrium obtained from the decidualized uterine horn of donor rats was finely minced and injected intraperitoneally into recipients with synchronized oestrus cycles using a 16G catheter (Persoons *et al.*, 2020). Here, a 1:1 ratio of donor to recipient tissue was used to induce the disease. Endometriosis-like lesions could be detected in 45% of the recipient animals after 12 weeks. Similar to the mouse model, unattached tissue can be observed in the abdominal cavity up to 12 weeks after seeding. The lesions varied in colour, abdominal location, and size. Notably, most of the lesions were located near connective tissue, intestines, or gonadal adipose tissue. Lesion size was considerably smaller than the suture model, and, in most cases, only one lesion could be retrieved. Interestingly, rats exhibit spontaneous postural changes that reflect ongoing pain behaviour, making it a useful model to study pain pathophysiology and potential treatments.

Alternatively, recipient Sprague-Dawley rats were injected intraperitoneally with pooled minced donor tissue (equivalent to one uterus) along the midventral line using an 18G needle (Cordaro *et al.*, 2021; D'Amico *et al.*, 2022a,b; Genovese *et al.*, 2021, 2022; Siracusa *et al.*, 2021a,b). The donor rats were synchronized by administering 10 IU PMSG. The rats were anaesthetized to harvest the uterus, ovary, and oviducts, and the uterine horn was minced in phosphate-buffered saline (PBS) prior to injection. Lesions were allowed to develop for 14 days, producing an average of two per recipient ∼1–1.5 mm in diameter. Disadvantages to the rat injection model include limitations on identifying the location of the lesions, the growth of the lesions, and minimal development of multiple lesions. The use of inbred strains would be expected to improve the number and duration of lesion implants.

### Endpoint assessment for mice and rats

To reduce the number of animals required to answer research objectives, we recommend that all studies include outcomes, lesion location, and lesion histology (see below) as well as several of the following endpoints. The inclusion of consistent outcomes will allow for harmonization across rodent endometriosis model studies and facilitate collaboration and hypothesis generation by cross-comparison. Bias should be minimized when making measurements. Rodents should be randomized to each experimental group, and the measuring investigator should be blinded to the experimental status of the animals as much as possible, with data analysis decisions (e.g. exclusion of outliers) being finalized before unblinding. Finally, appropriately powered sample sizes should be used and based on the variance, once known. The extent to which these procedures are employed must be made clear in both the laboratory notebook and the manuscript.

#### Lesion location

**Figure 2.**
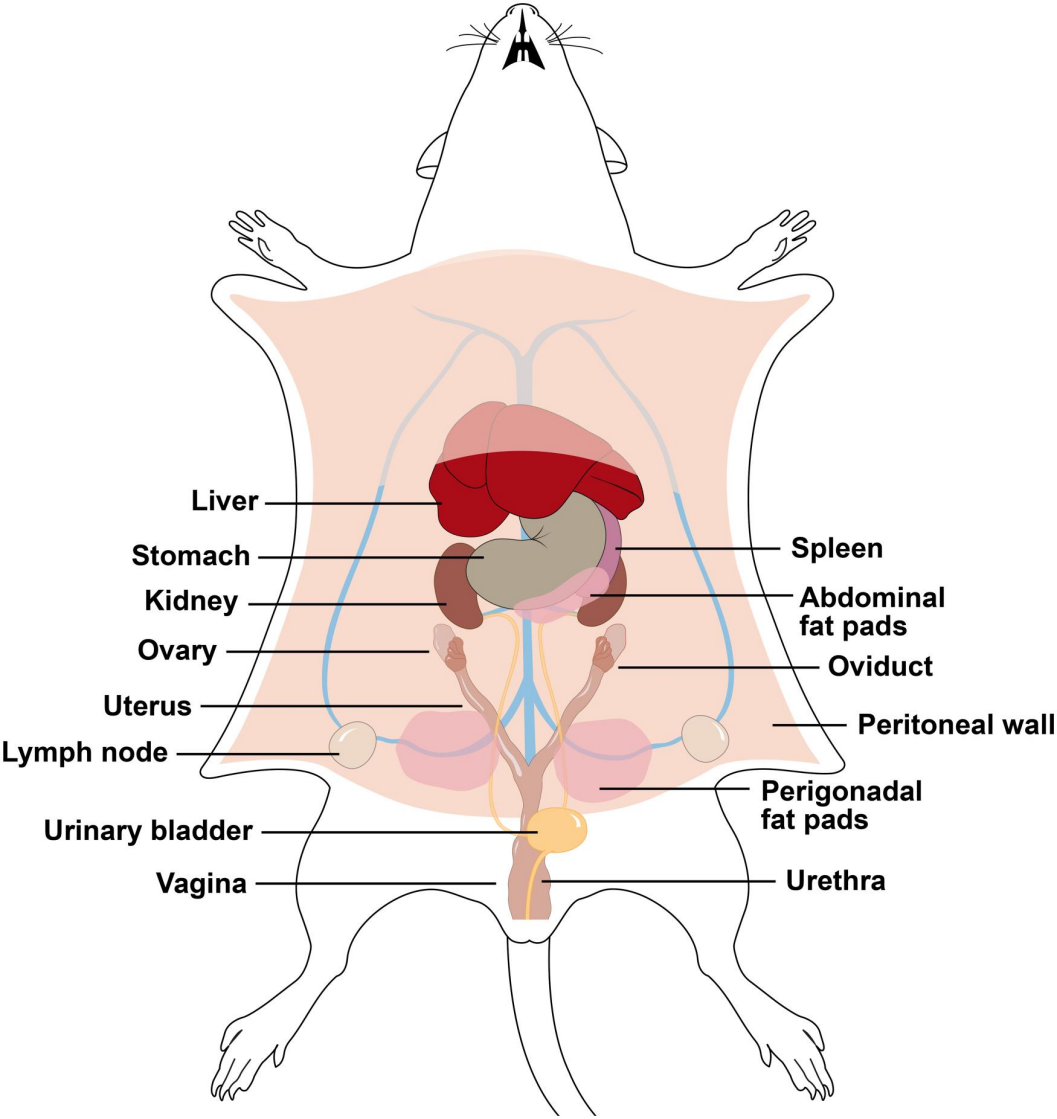
**Diagram for use in necropsy to locate endometriotic lesions.** The use of a diagram for each animal will allow for standardization of findings to indicate lesion location, size (weight and volume), colour, etc. The diagram can also be used to note other normal or abnormal findings (e.g. uterine weight, ovary weight, liver appearance). Intestinal and adipose tissues are not depicted.

Documentation of accurate lesion location is recommended. This documentation will allow investigators to capture lesion metrics for each animal. We provide a rodent body diagram that is helpful when marking lesion location in the peritoneal cavity (Fig. 2). On these sheets, for each animal, other metrics can be documented. We recommend the following: total lesion weight (each lesion alone may be too small to weigh), volume of each lesion as determined using callipers to take height, width, and length (mm^3^), lesion location and point of attachment, lesion colour, and presence of vascularization. Other important metrics include findings in the peritoneal cavity, such as the presence of adhesions, swollen lymph nodes, and/or the abnormal appearance of other organs.

#### Lesion histology

For any new model, the histology of lesions (e.g. H&E staining) must be carefully examined and verified in collaboration with an experienced endometriosis research scientist and/or pathologist with extensive experience in endometrial/endometriotic histology to determine the extent to which lesions resemble human endometriosis. Routine confirmation of lesion histology is advisable for all investigators but is especially important for new investigators in a laboratory, who may mistake other structures (e.g. lymph nodes or mammary tissue) for lesions.

#### Imaging

Fluorescence/bioluminescence imaging of lesions for localization *in vivo* allows for the longitudinal monitoring of the number and size of lesions as well as visualization of lesion location prior to necropsy, although this can be hampered by the length of the protocol and autofluorescence from gastrointestinal content (Dorning *et al.*, 2021). Photographic documentation of lesions using white light visualization at necropsy allows for lesion colour, approximate size, and localization, if imaged while in the animal. *Ex vivo* imaging will also enhance visualization for future resources and/or comparisons among laboratories.

#### Fertility

Endometriosis is known to adversely affect fertility and pregnancy outcomes in women; thus, assessment of these endpoints is appropriate for some studies (Macer and Taylor, 2012; Stilley *et al.*, 2012; Prescott *et al.*, 2016; Farland *et al.*, 2019; Breintoft *et al.*, 2021). Most fertility studies in rodents with endometriosis have been done in rat models of endometriosis (Vernon and Wilson, 1985; Barragan *et al.*, 1992; Moon *et al.*, 1993; Sharpe-Timms *et al.*, 1998; Sharpe-Timms, 2002; Stilley *et al.*, 2009; Stilley *et al.*, 2010; Stilley and Sharpe-Timms, 2012; Birt *et al.*, 2013a,b; Sharpe-Timms *et al.*, 2020; Kanellopoulos *et al.*, 2022). The homologous induction of endometriosis in rats has been used to characterize many defects that cause subfertility (Vernon and Wilson, 1985; Barragan *et al.*, 1992; Moon *et al.*, 1993; Sharpe-Timms *et al.*, 1998; Sharpe-Timms, 2002; Stilley *et al.*, 2009; Stilley *et al.*, 2010; Stilley and Sharpe-Timms, 2012; Birt *et al.*, 2013a,b; Sharpe-Timms *et al.*, 2020; Kanellopoulos *et al.*, 2022). In the earliest studies, endometrial tissue transplantation reduced both the number of pups at term (by 48%) and number of Day 14 embryos (by 28%). Several other studies confirmed lower pregnancy rates in endometriosis animals versus controls (Barragan *et al.*, 1992; Stilley *et al.*, 2010; Sharpe-Timms *et al.*, 2020). The endometriotic lesions have been shown to regress in pregnant rats (Vernon and Wilson, 1985; Rajkumar *et al.*, 1990) and during lactation demonstrating beneficial effects of anoestrous. This model also demonstrates anomalies of the hypothalamic–pituitary–ovarian axis, ovarian follicle development, and ovulatory dysfunction, including fewer ovarian follicles and corpora lutea with luteinized unruptured follicles (Moon *et al.*, 1993; Stilley *et al.*, 2010). Furthermore, *in vivo* anomalies in postovulatory oocyte structure and preimplantation embryo development, including misaligned chromosomes, nuclear and cytoplasmic fragmentation, and delayed or arrested cleavage, as well as lower implantation rates and spontaneous abortions were found in this model (Stilley *et al.*, 2009; Birt *et al.*, 2013b; Sharpe-Timms *et al.*, 2020). Emerging evidence demonstrates transgenerational impacts, leading to reduced fertility in females and males developmentally exposed to endometriosis over two and three generations (Birt *et al.*, 2013b, Sharpe-Timms *et al.*, 2020; Stilley *et al.*, 2009) A single fertility study in mice demonstrates that mice >13 weeks old with endometriosis had more resorbed foetuses, and the pups that were born were smaller than controls (Elsherbini *et al.*, 2022). No significant differences in fertility were seen between control and endometriosis in mice <7 weeks old (Elsherbini *et al.*, 2022). Given the current lack of studies and data exploring the impact of endometriosis on pregnancy outcomes in mice, our recommendations for fertility endpoints are to use the rat model of endometriosis and/or validate findings in mice.

#### Timing and development of endometriosis lesions

The time between disease induction and lesion harvesting in rodent lesions ranges from 24 h until 56 days with most studies examining lesions at 21 days (Burns *et al.*, 2021). Early timepoints (<24 h) are used to study angiogenesis, neurogenesis, and immune responses of the lesions during the early attachment and remodelling phases (Sanchez *et al.*, 2017; Burns *et al.*, 2021). Studies using unmodified endometrial tissue show lesion establishment at sites of attachment 24, 48, and 72 h after endometriosis initiation (Kobayashi *et al.*, 2011; Burns *et al.*, 2018). Gene expression at these stages indicates increased immune, angiogenic, and remodelling activity, independent of oestrogen/ESR1 signalling (Chen *et al.*, 2010; Burns *et al.*, 2018). By Day 7, lesion remodelling appears complete, with immune-related gene expression changes persisting at Days 7 and 14 (Panir *et al.*, 2024).

Beyond Day 7, studies focus on lesion growth, hormone responses, fibrosis, pain, infertility, and treatment efficacy (Duan *et al.*, 2018; Fattori *et al.*, 2020; Fattori *et al.*, 2024). A common approach involves lesion growth for 3-4 weeks, followed by a 3–4-week treatment period to assess (Soares *et al.*, 2012; Fattori *et al.*, 2020). For accurate treatment evaluation, lesions should persist for at least 6–8 weeks to confirm regression is due to therapy rather than natural resolution.

Inbred mouse strains maintain stable lesion numbers post-induction, with limited new lesion formation (Burns *et al.*, 2012; Jones *et al.*, 2018; Dorning *et al.*, 2021). Outbred strains develop fewer lesions, which regress over time. In humans, it has been postulated that endometriosis lesions may be able to seed new lesions, which could explain the recurrence of the disease post-hysterectomy (Rizk *et al.*, 2014; Praetorius *et al.*, 2022). Similarly, in baboons, lesions maybe dynamic, with lesions disappearing and appearing again over time (Harirchian *et al.*, 2012). Lesion size/volume has been shown to increase over time in the homologous mouse model (Duan *et al.*, 2018), mainly due to oestrogen-driven epithelial proliferation (Burns *et al.*, 2012; Liang *et al.*, 2018; Dorning *et al.*, 2021). Importantly, in the full thickness model, the lesions are cystic, and thus an increase in lesion volume can be contributed by an increase in cyst size with fluid secretion being a natural epithelial response to hormones and not to solid growth of the lesion tissue (Kobayashi *et al.*, 2011; Dodds *et al.*, 2017).

#### Pain

For pain behaviour assessment, metrics, and SOPs in rodent models of endometriosis, please refer to our EPHect companion paper (Dodds *et al.*, 2025).

### Control group selection

Identifying suitable control groups to compare findings from experimental models of endometriosis is critical to ensure statistical analyses and data interpretation are valid. As a general practice for rigour and reproducibility, controls should hold all conditions constant except the independent variable. Several options have been used in past animal studies on endometriosis and, as with selecting an appropriate model, ‘best-fit’ controls are dependent on the hypothesis being tested. For example, studies may evaluate rodents ‘with endometriosis’ versus ‘no endometriosis’. If inducing endometriosis via the intraperitoneal injection method, accompanying controls often receive intraperitoneal injection of vehicle alone (e.g. Dodds *et al.*, 2019; Fattori *et al.*, 2020). As mentioned above, some researchers alternatively inject non-endometrial tissue (e.g. Somigliana *et al.*, 1999; Morris *et al.*, 2021). For surgical induction of endometriosis, control animals undergo sham surgical procedures (e.g. Burns *et al.*, 2018). Using the autologous model as an example, this includes adding sutures (alone or with non-endometrial tissue) to the intestinal mesentery and mechanical manipulation of the uterine horn (e.g. Torres-Reveron *et al.*, 2016; Castro *et al.*, 2021). In both scenarios, all animals undergo identical procedures, though the control group does not receive donor endometrial tissue. Other studies may examine an ‘intervention’ versus ‘no intervention’ in their model of endometriosis. In these cases, the independent variable is a treatment. Endometriosis is therefore induced via the same method for all animals, with an experimental group receiving the treatment (i.e. a drug) and controls a placebo (i.e. vehicle alone) (e.g. Jones *et al.*, 2018; Hirakawa *et al.*, 2022; Muraoka *et al.*, 2023).

### Tissue collection, processing, and euthanasia

Tissue collection and processing are areas of standardization, but euthanasia is more dependent on local rules and regulations of local institutional, company, university, or government animal care and use protocols (Hubrecht and Carter, 2019). Most investigators, however, euthanize rodents with carbon dioxide. A second method of euthanasia, such as cervical dislocation, may be required.

For tissue collection, if animals are intact and cycling, we recommend euthanizing the rodents during the same stage of the oestrous cycle (i.e. oestrus) as genes/proteins in the lesions are often hormone responsive; therefore, ensuring a similar cycle stage will reduce unwanted variability due to hormonal differences (see Supplementary File S1: EPHect-EM-Homologous SOP 8 and 14). With cycling animals, obtaining the uterine weight adds an additional layer of confirmation of the staging along with evaluating the cytology of a vaginal lavage. For studies using treatments, ovarian and other organ weights can also be important metrics to document changes due to treatment.

Tissue processing can also be standardized (see Supplementary File S1: EPHect-HM-Homologous SOP 7 and 13). Once the animal is euthanized, the first step is to obtain peritoneal cavity cells from a lavage. In the rat, peritoneal fluid may be sufficiently abundant to be collected without lavage. Second, once the animal is opened, blood should immediately be collected, either from the descending aorta or via cardiac puncture. Obtaining the blood quickly ensures that clotting does not occur but also allows for a cleaner environment for lesion visualization, imaging, and descriptions (see Supplementary File S1: EPHect-EM-Homologous SOP 10). Rapid recovery of tissues is required, and tissues need to be stored on wet ice until they have been weighed, measured, and documented. When wanting to study gene expression, at least one lesion from each animal should be frozen for nucleic acid assessment and a second fixed for histology to confirm lesion structures. Lesions can be kept for DNA, RNA, and protein isolation by snap freezing in liquid nitrogen or on dry ice. Technologies for nucleic acid analysis (both RNA and DNA) from fixed tissues are becoming more commonplace but still require careful selection of methods, training, and controls to avoid artefactual data (Yong *et al.*, 2021). Fixing the lesions by formalin or paraformaldehyde allows for superior histological analysis to visualize glands, stroma, immune infiltrates, and fibrosis (compared to observations in frozen tissues). For fixed lesions, we recommend the collection of tissue adjacent to the lesion, which will allow visualization of invasion of surrounding tissues and whether adhesions are present. When freezing the lesions, we recommend removing extra tissues or planning for cell-type enrichment with microdissection to prevent contamination of non-lesion tissue in DNA, RNA, or protein results. For the other organs (e.g. kidney, spleen, etc.), if there are two, one can be fixed and one can be frozen. Alternatively, a small piece of organ or lesion, if large enough, can be cut off for fixing, with the rest being snap frozen.

## Discussion

Our knowledge of endometriosis and endometriosis-related animal models has advanced significantly in the last decade. However, differences in experimental design, execution, and analysis of studies using experimental models of endometriosis have contributed to discrepancies in the literature, resulting in an overall inability to replicate and validate research results. Having identified the unmet need to develop standards of practice in experimental models of endometriosis, the EPHect team convened a working group led by experts in rodent models for endometriosis. The goal of the working group was to develop standardized resources and recommendations to be used in endometriosis research when deploying experimental models to improve continuity, reduce variability, and ensure studies are properly controlled. This will facilitate comparisons across laboratory groups, as well as enable multi-centre collaborations. The resulting recommendations are deliberately kept open to ensure hypothesis-driven discovery-based science would not be hampered in the absence of a defined aetiology.

While standardization is advocated, we acknowledge that most models we evaluated are ‘generic’. This standardization, therefore, should not be construed to stifle innovation in establishing new models of endometriosis. We advocate that with each novel model developed, the recommended standardized documentation and reporting details are embraced to facilitate result dissemination, inference, and comparison. There are and will be rodent models of endometriosis that may better represent specific phenotypes and that can explore specific outcomes. For example, to investigate specifically, the effect of ovarian endometrioma on fertility, a targeted mouse model has been reported (Hayashi *et al.*, 2020).

**Figure 3.**
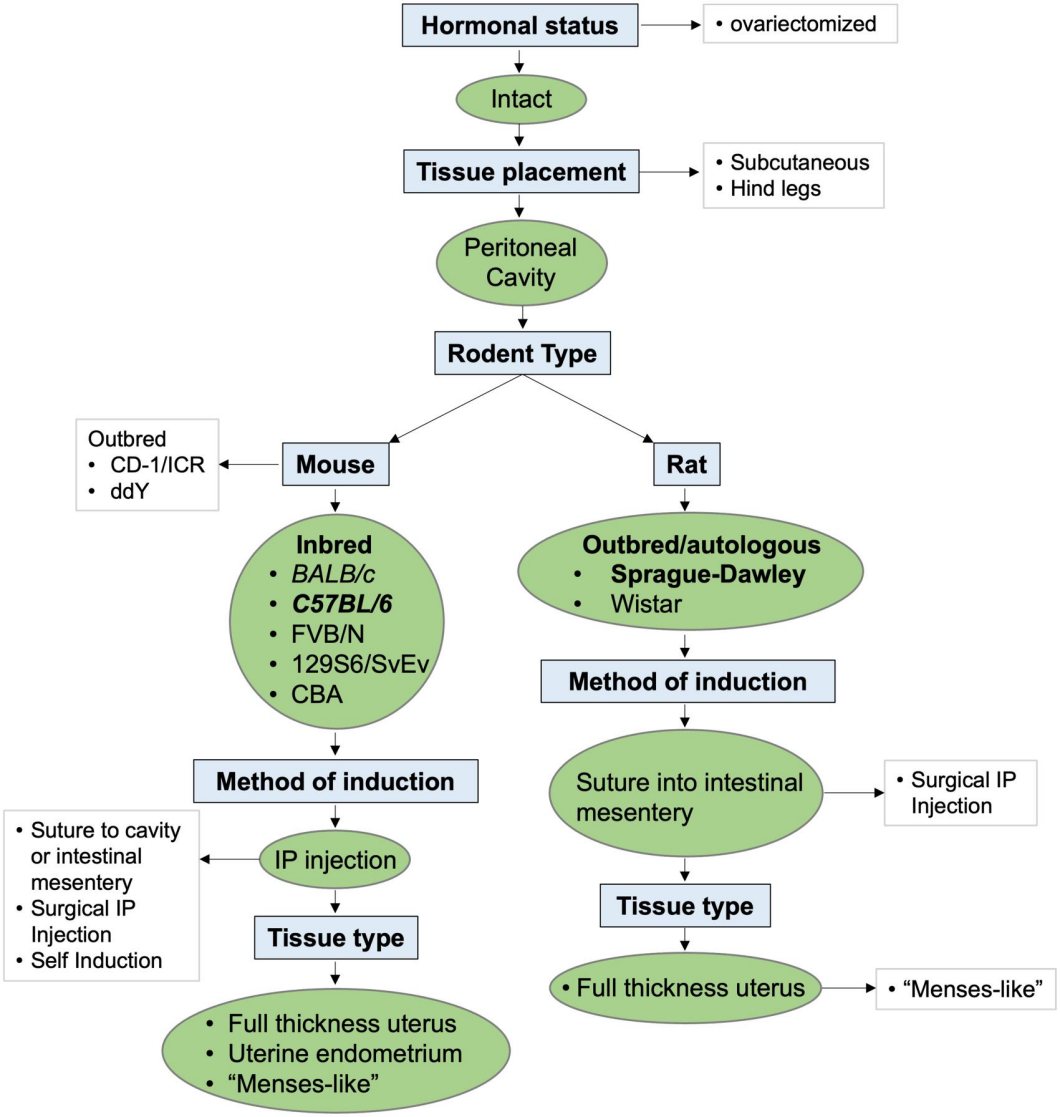
**Decision tree of key variables in homologous murine models of endometriosis.** The five main variables identified are shown in blue. Recommendations for mouse and rat are shown in green. IP, intraperitoneal; CD-1/ICR, Hsd: ICR (CD-19^®^); ddY, DDY/JcISidSeyFrkJ; FVB/N, Friend virus B NIH.

Derived from the advantages and disadvantages of each model, a decision tree was made to formalize our recommendations (Fig. 3). Our recommendations are also supported by the work of Nunez-Badinez *et al.* (2021), who found that currently, the most common homologous mouse model of endometriosis is the syngeneic intraperitoneal injection model. They also found that a high proportion of papers published used the autologous suture model of endometriosis, but this mouse model is no longer commonly used by laboratories (Nunez-Badinez *et al.*, 2021). Thus, like their findings, we too endorse the use of endometrium injected into the peritoneal cavity (wherever possible in ovary-intact animals) to develop endometriosis-like lesions in the mouse. The rat model, on the other hand, does not form robust lesions with intraperitoneal injection of uterine endometrium (Persoons *et al.*, 2020); therefore, the suture of size-defined full-thickness uterine tissue is recommended in the rat model.

Based on the myriad of commonalities among protocols received, we developed multiple SOPs to complement these recommendations for mice regarding pre- and post-operative care (Supplementary File S1: EPHect-EM-Homologous SOPs 1 and 10, respectively), injection of hormones (Supplementary File S1: EPHect-EM-Homologous SOPs 2 and 3), decidualization (Supplementary File S1: EPHect-EM-Homologous SOP 4), injection procedures (Supplementary File S1: EPHect-EM-Homologous SOPs 5 and 6), endpoint assessment and lesion collection (Supplementary File S1: EPHect-EM-Homologous-SOP 7), oestrous cycle staging (Supplementary File S1: EPHect-EM-Homologous SOP 8), and ovariectomy (Supplementary File S1: EPHect-EM-Homologous SOP 9). In a similar manner, SOPs were created for the rat model of endometriosis regarding pre- and post-operative care (Supplementary File S1: EPHect-EM-Homologous SOPs 11 and 15, respectively), induction of endometriosis (Supplementary File S1: EPHect-EM-Homologous SOP 12), endpoint assessment and lesion recovery (Supplementary File S1: EPHect-EM-Homologous SOP 13), and oestrous cycle staging (Supplementary File S1: EPHect-EM-Homologous SOP 14). In addition to the SOPs, to reduce variability, we also recommend detailed laboratory notebook keeping to record lesion location, volume, and colour, and other discernible characteristics (Fig. 2). In mice, we recommend at minimum that inbred mouse strains are used, uterine tissue is engrafted within the peritoneal cavity at a ratio of one donor mouse to two recipient mice, and exposure to oestrogens. Our standard recommendations are that C57BL/6 mice are used when possible, and that uterine tissue is engrafted via intra-peritoneal injection at a ratio of 1:1 in ovary-intact recipients (see Minimal and Standard Recommendations in Table 3). We envision that this will ensure data rigour, reproducibility, and repeatability from group to group. Recommendations are more simplistic for rat models, as less variation exists, but equally important to maintain replicability moving forward. Our standard recommendations are to auto-transplant 4–6 uterine tissue pieces onto the arterial cascades of the small intestine of ovary-intact Sprague-Dawley rats. We also recommend collecting a full repertoire of biospecimens and endpoint assessments in mice and rats to enable future collaborations or address new hypotheses whilst reducing the number of animals used in research. In studies that evaluate potential therapeutics, or aim to discover new therapeutic targets or biomarkers, we highly recommend the use of multiple pre-clinical models. This will test the accuracy/rigour of the discovery science in models that may represent different aetiologies. Regardless of the model decision made by individual groups, it will be imperative for both the research and peer reviewer community to encourage use of these standards and, at the very least, require complete and transparent reporting on the core variables described above. The resources and recommendations we have developed will aid in cross-institute collaboration for this exact purpose.

**Table 3.** EPHect-EM-homologous standard operating procedures: minimum and standard documentation for homologous rodent models.

Model Variable	Minimum	Standard	Notes
Mouse			
Species and animal strain	Inbred/syngeneic strain	C57BL/6	Outbred strains exhibit tissue rejection (unless auto-transplantation is performed).
Location of ectopic tissue placement	Intraperitoneal	Intraperitoneal	Other placements may be justified to answer specific research questions.
Volume of donor tissue	1 donor:2 recipient ratio	1 donor:1 recipient ratio (approximately 40 mg non-decidualized tissue)	
Method of induction	Suture	Injection	
Hormonal status of recipient	Supplementation with exogenous oestradiol if recipients are ovariectomized	Intact	Exogenous hormonal replacement(s) can answer specific research questions.
Donor uterine tissue ‘type’	Uterine tissue	Unable to standardize	The aetiology of endometriosis remains under investigation; thus, we do not recommend a ‘standard’ uterine tissue type to allow for innovation.
Rat			
Species and animal strain	Wistar	Sprague-Dawley	Both are outbred strains.
Location of ectopic tissue placement	Intraperitoneal	Sutured to the arterial cascades of the small intestine	Auto-transplantation is required due to use of outbred strains.
Volume of donor tissue	4 implants	4–6 implants	The same size uterine fragments should be used for each implant.
Method of induction	Suture	Suture	
Hormonal status of recipient	Supplementation with exogenous oestradiol if recipients are ovariectomized.	Intact	Exogenous hormonal replacement(s) can answer specific research questions.
Donor uterine tissue ‘type’	Uterine tissue	Unable to standardize	

We anticipate this EPHect initiative will inspire a new chapter in the standardization of endometriosis rodent model studies, including standardization of collection methods. Our recommendations on rodent models of endometriosis will inspire and foster new collaborations among existing endometriosis laboratory groups and encourage other investigators to join the field. This will allow us, as a community of researchers, to aid in the journey to understand the pathogenesis/aetiology of endometriosis that will, ultimately, be translated into improved treatments for the millions of those affected by this disease worldwide.

## Supplementary Material

gaaf021_Supplementary_Data

## Data Availability

No new data were generated or analysed in support of this research.

## Appendix: EPHect Experimental Models Working Group

Nick A. Andrews: Salk Institute for Biological Sciences, San Diego, CA, USA

Michael S. Anglesio: University of British Columbia, Vancouver, BC, Canada

Caroline B. Appleyard: Ponce Health Sciences University-Ponce Research Institute, Ponce, PR, USA

Joe Arosh: Texas A&M University, College Station, TX, USA

Christian M. Becker: University of Oxford, Oxford, UK

Kaylon L. Bruner-Tran: Vanderbilt University Medical Center, Nashville, TN, USA; World Endometriosis Research Foundation, London, UK

Katherine A. Burns: University of Cincinnati College of Medicine, Cincinnati, OH, USA

Ronald L. Chandler; Michigan State University College of Human Medicine, Grand Rapids MI, USA

Julie A. Christianson: University of Kansas Medical Center, Kansas City, MO, USA

Fiona L. Cousins: Hudson Institute, Clayton, VIC, Australia; Obstetrics and Gynaecology, Monash University, Clayton, VIC, Australia

Kelsi N. Dodds: Flinders University, Adelaide, SA, Australia; University of Adelaide, Adelaide, SA, Australia

Victor Fattori: Harvard University, Cambridge, MA, USA

Asgi Fazleabas: Michigan State University, Grand Rapids, MI, USA

Caroline Gargett: Hudson Institute of Medical Research, Clayton, VIC, Australia

Juan S. Gnecco: Tufts University, Medford, MA, USA

Raul Gomez; INCLIVA Instituto de Investigación Sanitaria, Valencia, Spain; University of Valencia, Valencia, Spain

Martin Götte: University of Münster, Münster, Germany

Erin Greaves: University of Warwick, Warwick, UK; World Endometriosis Research Foundation, London, UK

Linda G. Griffith: Massachusetts’s Institute of Technology, Cambridge, MA, USA

Patrick G. Groothuis: Byondis BV, Nijmegen, The Netherlands

Ruth Grümmer: University of Duisburg-Essen, Essen, Germany

Sun-Wei Guo: Fudan University, Shanghai, China

Shannon M. Hawkins: Indiana University School of Medicine, Indianapolis, IN, USA

M. Louise Hull: The Robinson Research Institute, University of Adelaide, Adelaide, SA, Australia

Lone Hummelshoj: World Endometriosis Research Foundation, London, UK

Mark R. Hutchinson: University of Adelaide, Adelaide, SA, Australia

Mohamed Gamal Ibrahim: Team Kinderwunsch Oldenburg GbR MVZ, Oldenburg, Germany

Elizabeth E. Marr: Tufts University, Medford, MA, USA

Stacy L. McAllister: Emory University School of Medicine, Atlanta, GA, USA

Stacey A. Missmer: Harvard TH Chan School of Public Health, Boston, MA, USA; University of Michigan, Ann Arbour (MI), USA; World Endometriosis Research Foundation, London, UK

Jeffrey S. Mogil: McGill University, Montreal (QC), Canada

Jens Nagel: Merz Therapeutics, Frankfurt, Germany

Warren B. Nothnick: Kansas University Medical Center, Kansas City, MO, USA

Paulina Nunez-Badinez: Bayer AG, Wuppertal, Germany (until 23 March 2023)

Kevin G. Osteen: Vanderbilty University Medical Center, Nashville, TN, USA

Daniëlle Peterse: Boston Childrens Hospital, Harvard Medical School, Boston, MA, USA

Michael S. Rogers: Boston Childrens Hospital, Harvard Medical School, Boston, MA, USA

Andrea Romano: Maastricht University, Maastricht, The Netherlands

Philippa T.K. Saunders: University of Edinburgh, Edinburgh, UK

Miguel Ángel Tejada: University of Granada, Granada, Spain

Kathy L. Sharpe-Timms: University of Missouri School of Medicine, Columbia, MO, USA

Waldiceu A. Verri: State University of Londrina, Londrina, Brazil

Paola Viganó: Fondazione IRCCS Ca’ Granda Ospedale Maggiore Policlinico, Milano, Italy

Katy Vincent: University of Oxford, Oxford, UK
