# Correction to: WERF Endometriosis Phenome and Biobanking Harmonisation Project for Experimental Models in Endometriosis Research (EPHect-EM-Homologous): homologous rodent models

**DOI:** 10.1093/molehr/gaaf036

**Published:** 2025-07-28

This is a correction to: Katherine A. Burns, Daniëlle Peterse, Caroline B. Appleyard, Ronald Chandler, Sun-Wei Guo, Amelia Pearson, Eleonora Persoons, Michael S. Anglesio, Michael S. Rogers, Kathy L. Sharpe-Timms, Joris Vriens, Stacy L. McAllister, Kelsi N. Dodds, Fiona L. Cousins, Lone Hummelshoj, Stacey A. Missmer, Kaylon L. Bruner-Tran, and Erin Greaves, for the EPHect Experimental Models Working Group, WERF Endometriosis Phenome and Biobanking Harmonisation Project for Experimental Models in Endometriosis Research (EPHect-EM-Homologous): homologous rodent models, *Molecular Human Reproduction* Volume 31, Issue 3, 2025; gaaf021, https://doi.org/10.1093/molehr/gaaf021.

In the original version of the article, there was a typographical error in the name of author Stacy L. McAllister in the author list. This has now been corrected.

