# Supplementary material for: WERF Endometriosis Phenome and Biobanking Harmonisation Project for Experimental Models in Endometriosis Research (EPHect-EM-Homologous): homologous rodent models
Source: Mol Hum Reprod. 2025 Jul 9;31(3):gaaf021. doi: 10.1093/molehr/gaaf021 (PMC12237519; doi:10.1093/molehr/gaaf021)
Supplement: gaaf021_Supplementary_Data [file gaaf021_supplementary_data.zip › MHR-24-0370.R1_Supplementary info.pdf]

Supplementary Information

World Endometriosis Research Foundation EPHect Experimental Models for Endometriosis Research (EPHect-EM-Homologous): homologous rodent models

Katherine A. Burns, Daniëlle Peterse, Caroline B. Appleyard, Ronald Chandler, Sun-Wei Guo, Amelia Pearson, Eleonora Persoons, Michael S. Anglesio, Michael S. Rogers, Kathy L. Sharpe-Timms, Joris Vriens, Stacey L. McAllister, Kelsi N. Dodds, Fiona L. Cousins, Lone Hummelshoj, Stacey A. Missmer, Kaylon L. Bruner-Tran, Erin Greaves, for the EPHect Experimental Models Working Group

Supplementary Figure S1: Flow diagram of the WERF EPHect-EM consortium's approach

Supplementary File S1 EPHect Standard Operating Procedure Experimental models: homologous (provided as a separate file)

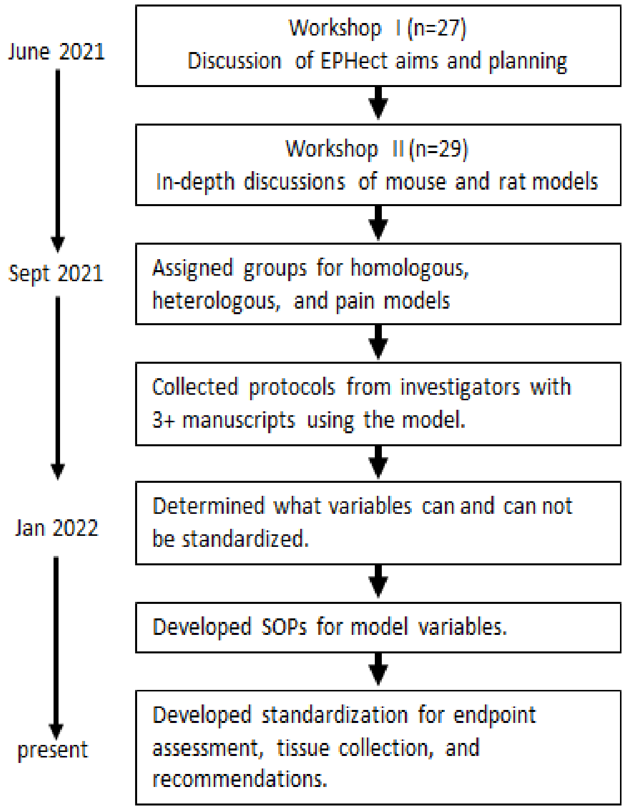

Supplementary Figure S1: Flow diagram of the WERF EPHect-EM consortium's approach. The flow diagram illustrates the systematic approach adopted by the WERF EPHect-EM consortium to standardise experimental models in endometriosis research. Key steps involved were: initial workshops and sharing of models and best practices with leading researchers in the field, collection of standard operating procedures (SOPs), harmonization of SOPs, and development of standardisation guidelines.
