# Supplementary material for: WERF Endometriosis Phenome and Biobanking Harmonisation Project for Experimental Models in Endometriosis Research (EPHect-EM-Homologous): homologous rodent models
Source: Mol Hum Reprod. 2025 Jul 9;31(3):gaaf021. doi: 10.1093/molehr/gaaf021 (PMC12237519; doi:10.1093/molehr/gaaf021)
Supplement: gaaf021_Supplementary_Data [file gaaf021_supplementary_data.zip › MHR-24-0370-R1-SuppFileS1.pdf]

Katherine A. Burns, Daniëlle Peterse, Caroline B. Appleyard, Ronald Chandler, Sun-Wei Guo, Amelia Pearson, Eleonora Persoons, Michael S. Anglesio, Michael S. Rogers, Kathy L. Sharpe-Timms, Joris Vriens, Stacey L. McAllister, Kelsi N. Dodds, Fiona L. Cousins, Lone Hummelshoj, Stacey A. Missmer, Kaylon L. Bruner-Tran, and Erin Greaves for the EPHect Experimental Models Working Group

TABLE of CONTENTS

Standardisation of homologous mouse models of experimental endometriosis 2
SOP 1: Pre-operative care 4
SOP 2: Hormonal injection of oestradiol benzoate and preparation of full thickness uterus 5
SOP 3: Hormonal injection of PMSG and preparation of uterine endometrium 7
SOP 4: Decidualization and preparation of endometrium 9
SOP 5: Induction of endometriosis in recipient mice via 18G needle 13
SOP 6: Induction of endometriosis in recipient mice via dorsal lateral incision 14
SOP 7: Endpoint assessment and collection of endometriosis lesions 15
SOP 8: Oestrous cycle staging 17
SOP 9: Ovariectomy 18
SOP 10: Post-operative care 19
Standardisation of homologous rat models of experimental endometriosis 20
SOP 11: Pre-operative care 22
SOP 12: Induction of endometriosis using full thickness uterus 23
SOP 13: Endpoint assessment and collection of endometriosis lesions 26
SOP 14: Oestrous cycle staging 28
SOP 15: Post-operative care 29

Standardisation of homologous mouse models of experimental endometriosis

Statement of purpose

The purpose of this standard operating procedure (Peterse *et al.*) is to describe techniques and methods most commonly used to model endometriosis-like lesions in **mice**. EPHect's purpose in the publication of these procedures is the standardisation (wherever possible, without limiting scientific creativity and discovery) of endometriosis experimentation across institutions to improve comparability of data, experimental integrity, and provide the mechanisms that lead to "best fit models" aiming to recapitulate human endometriosis pathophysiology.

Criteria

Endometriosis is a multi-system disease whose defining pathological feature is lesions, which are defined as immune infiltrated ectopic endometrial-like tissue, consisting of dense, endometrial-type stromal cells, glandular epithelial cells, the presence of hemosiderin and accumulation of extracellular matrix consistent with fibrosis. For the basis of any current or future model, the endometriosis-like lesions need to meet these criteria with the recognition that lesion histology is very diverse and that on occasions, endometriosis lesions might be harvested that do not contain all of the above-mentioned hallmarks.

Recommendations

A number of recommendations were made to begin to harmonize how rodent studies on endometriosis are performed and collected. Standards and forms were developed for the model variables (see SOPs below). Overall, most commonly, studies begin with animals in the age range of 8-12 weeks. At surgery, if surgery is performed, most protocols used buprenorphine for pain control. This should be discussed with individual animal units and vets to ensure compliance and mitigate pain in animals, although we recognise that availability in some locations may be limited. Pain management immediately post-surgery is recommended, however we suggest avoiding or minimising the use of analgesic agents with anti-inflammatory actions (opioids, non-steroidal anti-inflammatory (NSAIDs), and others) to mitigate potential confounding inflammatory processes that are features of endometriosis. When anesthetising the animals, isoflurane and oxygen is most used with ketamine being used for lengthier surgeries (i.e., laparoscopy).

To standardise studies more formally across various aspects of science and disease, general practices should include power calculations to determine the number of animals needed for proper statistical analysis, randomization of animals and samples when processing, and to follow the Animal Research: Reporting of *In Vivo* Experiments (ARRIVE) guidelines set by the National Centre for the Replacement, Refinement, and Reduction of Animals in Research (Percie du Sert *et al.*, 2020a, Percie du Sert *et al.* , 2020b). All these general practices strongly complement the recommendations made by EPHect for the use of rodent models of endometriosis.

Key model variables and recommendations were categorized for homologous **mouse** models of endometriosis as follows:

1. Strain: Syngeneic inbred mice are recommended and preferred due to immunological acceptance of tissue. Most commonly, the C57BL/6 mouse strain (8 - 12 weeks at the start of the experiment) is used. C57BL/6 are used because the majority of genetically modified mice are available on this background. BALB/c mice are also a common option as this strain of mice has a more reliable baseline behaviour and response to pain than C57BL/6 strain (see WERF companion paper Dodds *et al*., tbc).
2. Uterine donor tissue 'type': The aetiology of endometriosis remains unknown; thus, we are unable to make a recommendation of the 'type' of uterine tissue used to establish experimental lesions. Not standardizing this variable preserves the ability of the models to evolve alongside scientific knowledge and advancements. However, all models use displacement of uterine tissue to generate lesions. Currently several variations exist that use: full thickness uterus including myometrium, endometrium with myometrium removed, as well as 'menses'-like

endometrial tissue. For studies interrogating the performance of potential therapeutics our recommendation would be to utilise multiple models for robust testing (may require collaboration with other groups).

3. Method of induction: Injection method is preferred either via needle/trocar or incision.
4. Placement of tissue: Peritoneal cavity placement is recommended.
5. Amount of tissue: One uterus to one recipient mouse which equates to about 40 mg of tissue. When using decidualized tissue, the amount of tissue from one uterine horn can be between 40-100 mg.
6. Hormonal status of recipient: Ovary-intact mice are recommended. Minor differences in lesion attachment are observed when tissue is injected into the recipient at specific oestrous stages; therefore, some researchers may stage the mice at disease initiation (donor or recipients). Some researchers prefer to ovariectomize recipients and add back oestradiol, although it is documented that surgery and hormone replacement alters the immune environment and can affect pain responses (see WERF companion paper Dodds *et al.*, tbc).
7. Endpoint assessment and collection of endometriosis lesions: Experimental end points should be hypothesis driven. Collection of multiple endpoint assessments will allow for improved study comparability. At least a fraction of lesions should be routinely collected and microscopically examined to confirm endometriosis-like histology. We strongly encourage researchers to adhere to Animal Research: Reporting of *in vivo* Experiments (ARRIVE) guidelines.

The following SOPs are included in this document

Pre- and post-operative care

- SOP 1: Pre-operative care
- SOP 10: Post-operative care

Procedures for uterine preparation and harvest

These are the least standardizable SOPs for the homologous mouse model of endometriosis. Multiple methods are used to prepare uterine tissue for developing endometriosis lesions. The three most common are described below:

- SOP 2: Hormonal injection of oestradiol benzoate and preparation of full thickness uterus
- SOP 3: Hormonal injection of pregnant mare serum gonadotrophin (PMSG) and preparation of endometrium
- SOP 4: Decidualization and preparation of endometrial tissue

Procedures for the induction of endometriosis

The most common method to induce endometriosis lesions is by injection. Two methods are commonly used.

- SOP 5: Injection with 18G needle or trocar
- SOP 6: Incision into the peritoneal cavity

Endpoint assessment and collection of endometriosis 'lesions'

- SOP 7: Endpoint assessment and collection of endometriosis lesions

Oestrous cycle staging

- SOP 8: Vaginal cytology in lavages

Ovariectomy

- SOP 9: Ovariectomy

REFERENCES

Percie du Sert N, Ahluwalia A, Alam S, Avey MT, Baker M, Browne WJ, Clark A, Cuthill IC, Dirnagl U, Emerson M *et al.* Reporting animal research: Explanation and elaboration for the ARRIVE guidelines 2.0. *PLoS Biol* 2020a;18:e3000411.

Percie du Sert N, Hurst V, Ahluwalia A, Alam S, Avey MT, Baker M, Browne WJ, Clark A, Cuthill IC, Dirnagl U *et al.* The ARRIVE guidelines 2.0: Updated guidelines for reporting animal research. *PLoS Biol* 2020b;18:e3000410.

SOP 1: Pre-operative care

Purpose
To prepare rodents for surgery. Prior to conducting any surgical procedures, mice are acclimated (if received from a supplier or other unit), given a health check, and weighed. Surgery should be scheduled early in the day and conducted at the beginning of the week to allow for adequate monitoring of animals. All instruments should be sterilized before use and can be sterilized between animals using a bead sterilizer or cold sterilant.

PROTOCOL
1. Sterile surgical gloves must be worn. All areas should be cleaned with a sterilizing agent (e.g. Trigene wipes™ or Super Sani-Cloth®) including heating pad/blanket where possible.
2. Place sterile instruments and tools on a sterile surface (e.g. autoclaved metal tray, a sterile drape or towel) during the procedure.
3. Weigh and anaesthetise animal using inhalation anaesthesia following the appropriate (facility-specific) SOP.
4. Check anaesthetic depth by measuring withdrawal and eye position reflexes.

   ***Note:** In rodents, withdrawal reflexes are tested by pinching the tail and the hind limbs to demonstrate the reflex is absent or barely detectable. The eye becomes fixed following anaesthetic induction, and the blink reflex should be absent. A lubricating eye ointment, like Lacri-lube®, can be placed in the eyes whenever anaesthetics are used to prevent physical damage and dryness.*

5. Following anaesthesia induction, carefully monitor and support body temperature, heart, and respiratory rates. Place rodent on a heating pad for the duration of the procedure**.** Monitor respiratory rates.

   ***Note**: Small animals have a high surface area to body weight ratio and lose heat rapidly. As most anaesthetics depress thermoregulation, hypothermia can develop rapidly, so rodents should be kept on a heat pad for the duration of the procedure to maintain a body temperature between 35-37°C. Ensure all fluids to be administered are warmed to 35 - 37°C before administration. Respiratory rate should be maintained at 40-100 breaths per minute (bpm) for rodents.*

6. Remove hair from the surgical site (caudal dorsal position) with clippers (approximately 1.5 x 1.5 cm square area).

   ***Note:** If possible, animal preparation should be in a different area from the surgical area. Leave sufficient margin to ensure the hair does not enter the wound but avoid over clipping as this will remove insulation. Surgical gloves should be changed or hair removal done by a separate person to maintain sterility.*

7. Once correct anaesthetic depth is achieved, transfer animal to a nose cone for surgery. Re-check anaesthetic depth. We recommend that analgesia be given prior to surgery by subcutaneous injection into the interscapular region.

   ***Note:** Buprenorphine (0.05 - 0.1 mg/kg in 100 µl) (an opioid derivative) is recommended for pain relief in models of inflammation as anti-inflammatory drugs (such as NSAIDS) may interfere with the inflammatory component of the model and may compromise the effectiveness of the procedure.*

8. Position animal as required in the surgical area, on top of a heating pad and disinfect the skin by applying a disinfectant solution (e.g. Hibitane). Apply a suitable drape.

SOP 2: Hormonal injection of oestradiol benzoate and preparation of full thickness uterus

Purpose

A prevailing hypothesis for the development of endometriotic lesions in women is via retrograde menstruation. During menstruation, the endometrium of the uterus is shed. Mice do not menstruate but exhibit hormonal patterns similar to humans in a much shorter period (i.e. 4-5 days). SOPs 2 4 describe the different methods used by researchers to prepare full thickness / endometrium only for injection into the peritoneal cavity of recipient mice.

In SOP 2 uterine pieces from a donor animal are implanted into the peritoneal cavity of a recipient mouse. To optimize the amount to donor tissue, animals are injected with 3 µg oestradiol benzoate (long acting), which stimulates uterine growth and results in a larger volume of tissue to harvest.

Note

Procedures are thoroughly described in Fattori, 2020 (Fattori *et al.*, 2020). Oestradiol benzoate is dissolved in DMSO at a concentration of 3 mg/ml. Next this solution is diluted 100x in sesame oil immediately prior injection to a final concentration of 30 µg/ml. 100 µl is injected subcutaneously (SC) into each donor mouse one week prior to (day -7) endometriosis induction.

PROTOCOL

A. Oestradiol preparation

Note*: Oestradiol benzoate is dissolved in DMSO as a stock solution. A fresh working solution is prepared using sesame oil right before injection into the donor mice.*

1. Weigh 3 mg of oestradiol benzoate and place into a 1.5 mL microcentrifuge vial.
2. Add 1 mL of DMSO and invert several times until dissolved. If little particles remain, vortex. Stock solution remains stable for several months frozen at 4°C or -20°C.
3. Immediately prior to injecting donor animals: For a cohort with 10 donor mice, 990 µl sesame oil is placed in a microcentrifuge tube and 10 µl of the stock solution is added (30 µg/ml final concentration). Note working solution should be prepared according to amount of donor mice for each cohort. Vortex mixture to ensure equal concentration throughout vial.

B. Hormonal injection

Note: *The oestradiol working solution (prepared in step 1) is subcutaneously injected into donor mice 7 days before harvesting uterus.*

1. Inject 100 µl per mouse using a 25G needle plus syringe.
2. Scruff mouse and inject subcutaneously by placing needle in the interscapular region of the animal.

C. Uterine harvest of donor mice

Note: *Following euthanasia, uterus of the donor mouse is removed en bloc.*

1. Euthanize mice by an approved/appropriate method and confirm death (most commonly used method is isoflurane followed by cervical dislocation as confirmation of death).
2. Make incision in lower abdomen and separate skin from peritoneal cavity using blunt dissection.
3. Pull skin away to deglove and expose the peritoneal wall of the mouse.

4. Open peritoneal wall and move intestines and uterine fat pad aside to reveal uterus.
5. Grasp uterus at midpoint and cut above cervix.
6. Run slightly closed scissors down each uterine horn to remove excess fat and cut right before the oviduct/ovary.
7. Place uteri in sterile PBS/HBSS and bring to clean area. Solutions are kept at room temperature.

D. Full thickness uterine tissue preparation (day 0)

Note: *The donor mouse is anesthetized and sacrificed after which the uterus is dissected, cut into small fragments and will be used to induce endometriosis in a recipient mouse (1 donor mouse to 1 recipient mouse or 1 donor mouse to 2 recipients can be used.)*

1. Place the uterus in a 10 cm petri dish containing 1-2 mL sterile PBS/HBSS to wash the uterus
2. Place uterus into a new petri dish containing 500 µl HBSS. Using a scalpel and forceps, cut the uterus into small fragments (<1 mm). If desired, uterine horn can first be cut open using forceps and small scissors. Take care not to cut and include plastic as this creates an inflammatory response.
3. Draw up small fragments and liquid into a 1 ml syringe. Final volume should be 500 µl.
4. Place an 18G needle on top of the syringe to prepare for injection.

For procedures for the induction of endometriosis see SOP 5 and 6.

REFERENCE

Fattori V, Franklin NS, Gonzalez-Cano R, Peterse D, Ghalali A, Madrian E, Verri WA, Jr., Andrews N, Woolf CJ, Rogers MS. Nonsurgical mouse model of endometriosis-associated pain that responds to clinically active drugs. *Pain* 2020;161:1321-1331.

SOP 3: Hormonal injection of pregnant mare serum gonadotrophin (PMSG) and preparation of uterine endometrium

Purpose

To collect endometrium during the low hormone stage, mice are primed with low dose pregnant mare serum gonadotropin (PMSG) 3.3-3.5 IU approximately 41 hours prior to harvest. Vaginal cytology from lavages of mice is examined at 41 hours to assess dioestrus to proestrus cycle stage. Transplantation of endometrium stripped of excess tissue, myometrium, and fat is transplanted into recipient to recapitulate endometriosis experimentally (Burns *et al.*, 2022).

Note

These procedures are described by Jones 2018 (Jones *et al.*, 2018), Burns 2017 (Burns *et al.*, 2017), and Burns 2012 (Burns *et al.*, 2012). Uterine harvest is performed on mature female mice above 6 weeks of age. Donor mice are primed with PMSG 41 hours prior to uterine harvest. PMSG triggers superovulation in mice and has the effect of synchronizing oestrous cycles in a group for a short period of time. By waiting 41 hours after PMSG injection, donor mice vaginal lavage will be in dioestrus-proestrus. After euthanasia, the uterus is removed *en bloc* and stripped of its excess tissue, fat, and myometrial layer before being minced and suspended in PBS.

PROTOCOL

A. Priming donor mice (-41 hours)

Note: *Donor adult female mice are primed 41 h before uterine harvest with PMSG.*

1. PMSG is purchased in 10,000 IU vials. Store at -20°C until use.
2. Resuspend PMSG with 1000 µl of sterile water and vortex. Once resuspended, make thirty (30) 1.5 mL tubes and aliquot 32.5 µl per tube. Keep on ice during aliquoting and store at -80°C until use.
3. Thaw PMSG aliquot and add 967 µL 0.9% sterile saline.
4. Inject mice intraperitoneally with 100 µL PMSG 41 hours prior (about 5:00 pm) to harvest for mice to receive a dose of 3.3 IU of PMSG.

B. Uterine harvest (day 0)

Note: *Following euthanasia, donor uterus is removed en bloc.*

1. Mice should be euthanized by an approved/appropriate method and death confirmed (most commonly used method is isoflurane followed by cervical dislocation as confirmation of death). Two mice are euthanized at the same time.
2. Make incision in the lower abdomen and separate skin from peritoneal cavity.
3. Pull skin away to deglove mouse and reveal peritoneal wall.
4. Open peritoneal wall and move the intestines and uterine fat pad aside to reveal uterus.
5. Grasp uterus with forceps at the midpoint and using scissors, cut above cervix.
6. Run slightly closed scissors down each uterine horn to remove excess fat and cut right before the oviduct/ovary.
7. Place uteri in sterile PBS and bring to sterile area.

C. Uterine tissue preparation (day 0)

***Note:** In a sterile area, uteri are washed 2x more with PBS, uteri are then cleaned of excess tissue and outer myometrial layers are carefully peeled away. One uterine horn from each mouse is taken and combined with another horn for two full uterine horns. Uterus is minced into < 1.5 mm pieces for injection.*

1. With fine tipped forceps (RS-5095, Roboz), remove excess tissue and outer myometrial layers. Typically, uterus is held in place with one set of forceps and another pair is used to slowly and carefully peel away each layer. The myometrial layer will resemble muscle and be somewhat fibrous when it peels away.
2. Two uterine horns, one from each mouse, are combined to reduce variability and standardize across animals.
3. Once both horns are cleaned of myometrium, rinse in sterile PBS and splay open uterine horns longitudinally (RS-5650; Roboz).
4. In a glass petri dish or similar, using micro dissecting scissors (RS-5906SC, Roboz), mince uterus into ≤ 1.5 mm pieces (typically approximately 80 minces while moving tissue around) and resuspend in 500 µL sterile PBS in a 1000 µL pipet for injection. Typically, a razor blade is used to cut off the tip of the pipet tip for injection via incision. Pipet tips are pre cut and autoclaved prior to surgery.

***Note:** A plastic petri dish is not advised as mincing may introduce plastic into the tissue and create an inflammatory response.*

For procedures for the induction of endometriosis see SOP 5 and 6.

REFERENCES

Burns KA, Pearson AM, Slack JL, Por ED, Scribner AN, Eti NA, Burney RO. Endometriosis in the Mouse: Challenges and Progress Toward a 'Best Fit' Murine Model. *Frontiers in Physiology* 2022;12.

Burns KA, Rodriguez KF, Hewitt SC, Janardhan KS, Young SL, Korach KS. Role of Estrogen Receptor Signaling Required for Endometriosis-Like Lesion Establishment in a Mouse Model. *Endocrinology* 2012;153:3960-3971.

Jones RL, Lang SA, Kendziorski JA, Greene AD, Burns KA. Use of a Mouse Model of Experimentally Induced Endometriosis to Evaluate and Compare the Effects of Bisphenol A and Bisphenol AF Exposure. *Environmental Health Perspectives* 2018;126:127004.

SOP 4: Decidualization and preparation of endometrium

Purpose

To artificially induce a 'menses-like' event of endometrial breakdown and shedding.

Note

Mice do not menstruate; unlike the human endometrium, mouse endometrium does not spontaneously decidualise rather it requires the presence of a blastocyst to stimulate decidualisation of the upper functional layer in preparation for pregnancy. If a pregnancy is not established, the decidua will be reabsorbed, and the uterus will be remodelled during the oestrous cycle. In contrast, in humans and some primates the decidua is shed from the uterus during menstruation and the uterus subsequently undergoes repair and remodelling. This mouse model of menstruation therefore aims to artificially induce menstruation in mice in order to study human menstrual physiology in more detail. This model is also used to generate donor endometrial tissue that can be collected and introduced into recipient mice to form lesions shown to be histologically and morphologically similar to lesions found in human cases of endometriosis, therefore presenting a clinically relevant disease model.

These procedures are described by Cousins et al (Cousins *et al.*, 2020; Cousins *et al.*, 2014), Greaves *et al* (Dorning *et al.*, 2021; Greaves *et al.*, 2014a; Greaves *et al.* 2014b; Greaves *et al.*, 2017; Greaves *et al.*, 2015) and Peterse *et al* (Peterse *et al.*, 2018a; Peterse *et al.*, 2018b; Peterse *et al.*, 2024). The menses models are performed on mature female mice over the age of 8 weeks. See Figure 1 for the 19-day menses procedural timeline. Hormone injections should be performed early in the morning, decidualization is performed approximately four hours after the final 5 ng oestradiol (E) injection, and the tissue collection occurs approximately 4 - 6 hours after removal of the progesterone pellet.

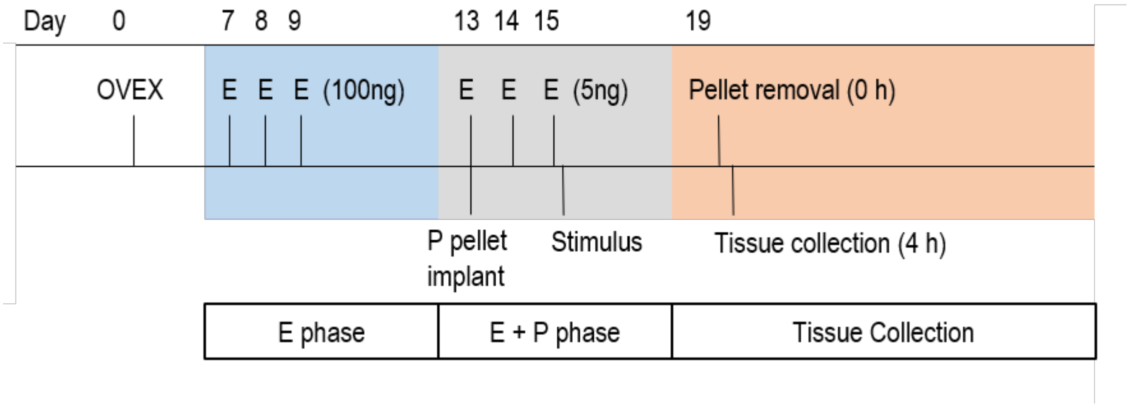

Figure 1. 'Menses' model: *Female mice are ovariectomised (OVEX), allowed to recover for 7 days and to clear endogenous hormones before giving subcutaneous (SC) injections of β-oestradiol E2 (E) for three consecutive days. A 3-day break is included followed by SC insertion of a progesterone implant (P) and 3 more days of E injections. Decidualization stimulus is carried out by injecting 20 µl of sesame oil into the uterus via the cervix using a non-surgical embryo transfer device, 4 hours after the final E injection (Stimulus). After another 3-day break, the P implant is removed to induce a progesterone withdrawal bleed. After 4 hours the donor mice are culled and uterus is removed for analysis or transfer into recipient mice. Adapted from (Cousins et al., 2014)*

PROTOCOL

A. Pre-operative care (see SOP1)

B. Ovariectomy (day 0) of mice (see SOP9)

C. Induction of 'menses' in mice

Injection of 17β-oestradiol (E2)

Note: *Seven (7) days following OVEX, administer E2 for 3 consecutive days*

1. Dilute E2 (from stock made up in DMSO) in sesame oil to 100 ng/100 µl.
2. On days 7, 8, and 9, mice should be SC injected with 100 µl of diluted E2.

P4 implant insertion

Note: *Following a further period of rest for 3 days, P4 implant is inserted for 6 days (day 13). Pre-operative care should be followed as described in SOP 1.*

1. Make a midline incision in skin between shoulder blades using sharp scissors.
2. Gently separate skin from underlying fascia to create a 'pocket' to hold P4 implant (bought or made*).
3. Place P4 implant in 'pocket' and close wound with wound clips or suture.

Note: ** SILASTIC (Dow Corning) implants can be made from laboratory tubing (1.2 cm in length, 1 cm usable length, 0.1 cm for sealing either end) filled with progesterone powder. A serum progesterone concentration of 8ng/ml can be expected.*
Post-operative care should be followed as described in SOP10.

Injection of E2

Note: *Following progesterone, administer E2 for 3 consecutive days (days 13, 14, and 15).*

1. Dilute stock E2 (made up in DMSO) in sesame oil to 5 ng/100 µl.
2. On days 13, 14, and 15, subcutaneously inject donor mice with 100 µl of diluted E2.

Decidualisation of mice

Note: *Decidualisation is induced approximately 4 hours after final 5 ng E2 injection on day 15. Pre-operative care should be followed as described above. A new non-surgical embryo transfer (NSET^{TM}; ParaTechs) device should be used for each animal. Alternatively, gel loading tips or blunt needles may be effective and have been used by some groups.*

1. Place the NSET device onto p20 pipette and load with 20 µl heat inactivated sesame/peanut oil.
2. Gently ease the smaller speculum into the vagina.
3. Gently insert the NSET device into the vagina and through the cervix.
4. Expel oil slowly, withdraw NSET device and remove speculum.

***Note**: Post-operative care should be followed as described above.*

Implant removal from mice (day 19)

***Note**: Four days after decidualisation, remove P4 implant to induce progesterone withdrawal bleed. Pre-operative care should be followed as described above.*

1. Carefully remove wound clip from incision.
2. Using a sterile scalpel, open incision and gently ease progesterone P4 implant from 'pocket'.
3. Close wound with wound clip or suture.

***Note:** Post-operative care should be followed as described above (SOP10).*

D. 'Menses-like' tissue collection from mice

***Note:** Four to six (4-6)** hours after removal of P4 pellet, menses-like endometrium can be collected. Mice should be euthanized with approved/appropriate method and death confirmed (most commonly used method is CO_2 followed by cervical dislocation as confirmation of death).*
****** This time could vary, but publications to date have used 4-6 hours post P4 withdrawal.*

1. The uterus should be removed alongside any other tissues of interest for analysis.
2. Prepare 'menses' material from donor mouse so that an 18G needle is loaded with approximately 40 – 100 mg decidualised endometrium from one donor uterine horn to a volume of 200 µl in sterile saline.

At this point the menses protocol is complete*,* if continuing with the endometriosis model follow SOPs for endometriosis-like lesion induction.

For procedures for the induction of endometriosis see SOP 5 and 6.

REFERENCES

Cousins FL, Murray A, Esnal A, Gibson DA, Critchley HO, Saunders PT. Evidence from a mouse model that epithelial cell migration and mesenchymal-epithelial transition contribute to rapid restoration of uterine tissue integrity during menstruation. *PLoS One* 2014;9:e86378.

Cousins FL, Farley JK, Kerrigan R, Mukherjee S, Darzi S, Gargett CE, Deane JA. The effects of hedgehog ligand neutralising antibody 5E1 in a mouse model of endometriosis. *BMC Res Notes* 2020;13:454.

Greaves E, Collins F, Esnal-Zufiaurre A, Giakoumelou S, Horne AW, Saunders PTK. Estrogen Receptor (ER) Agonists Differentially Regulate Neuroangiogenesis in Peritoneal Endometriosis via the Repellent Factor SLIT3. *Endocrinology* 2014a;155:4015-4026.

Greaves E, Cousins FL, Murray A, Esnal-Zufiaurre A, Fassbender A, Horne AW, Saunders PT. A novel mouse model of endometriosis mimics human phenotype and reveals insights into the inflammatory contribution of shed endometrium. *Am J Pathol* 2014b;184:1930-1939.

Greaves E, Horne AW, Jerina H, Mikolajczak M, Hilferty L, Mitchell R, Fleetwood-Walker SM, Saunders PT. EP(2) receptor antagonism reduces peripheral and central hyperalgesia in a preclinical mouse model of endometriosis. *Sci Rep* 2017;7:44169.

Greaves E, Temp J, Esnal-Zufiurre A, Mechsner S, Horne AW, Saunders PT. Estradiol is a critical mediator of macrophage-nerve cross talk in peritoneal endometriosis. *Am J Pathol* 2015;185:2286-2297.

Peterse D, Binda MM, O DF, Vanhie A, Fassbender A, Vriens J, D'Hooghe TM. Of Mice and Women: A Laparoscopic Mouse Model for Endometriosis. *J Minim Invasive Gynecol* 2018a;25:578-579.

Peterse D, Clercq K, Goossens C, Binda MM, F OD, Saunders P, Vriens J, Fassbender A, D'Hooghe TM. Optimization of Endometrial Decidualization in the Menstruating Mouse Model for Preclinical Endometriosis Research. *Reprod Sci* 2018b;25:1577-1588.

Peterse D, Verhassel A, Fassbender A, O DF, Vanhie A, Rompuy A-SV, Saunders P, Vriens J, D'Hooghe T. Investigating endometriosis development and endometriosis-related pain over time and in relation to estrogen in a laparoscopic mouse model. *bioRxiv* 2024:2024.2003.2028.583957.

SOP 5: Induction of endometriosis in recipient mice via 18G needle

Purpose
To dissemination uterine tissue into the peritoneal cavity for spontaneous formation of lesions.

PROTOCOL

A. Prepare uterine material

1. Prepare uterine material from donor mouse (Mouse-Homologous-SOP 2, 3, or 4). Donor material should be homogenized such that it can pass smoothly thorough an 18G needle. An 18G needle/syringe is loaded with donor tissue (40-100 mg) to a volume of 200-500 µl in sterile saline.
2. Draw back the plunger such that an air bubble is introduced between the end of the plunger and the tissue (this will ensure all the tissue is expelled during injection into the peritoneal cavity).

B. Inject uterine tissue into the peritoneal cavity

Note: *Pre-operative care should be followed as described in SOP1 if the animal is to be anesthetised during this step. Some facilities may not require analgesia at this step unless discomfort is observed; some researchers recommend that no analgesia is preferential where compliant with care regulations at this step.*

1. Swab around injection site with surgical scrub solution.
2. If the animal is anesthetized, take hold of the skin at umbilicus with forceps and pull away from internal organs. In not, the animal is scuffed for restraint.
3. Inject 200-500 µl of endometrial tissue into peritoneal cavity of recipient mouse and gently massage to disperse tissue fragments.

Note*: If the animal is anesthetized post-operative care should be followed as described in SOP10.*

SOP 6: Induction of endometriosis in recipient mice via dorsal lateral incision

Purpose

To dissemination uterine tissue into the peritoneal cavity for spontaneous formation of lesions.

PROTOCOL

A. Prepare uterine material

1. Prepare autoclaved p1000 tips with about 1.2 cm cut off the tip.
2. Prepare uterine material from donor mouse (SOP 2, 3, or 4). Donor material should be finely minced (≤1.5 mm).
3. Resuspend donor uterine tissue in 200 - 500 µL in PBS.

B. Inject uterine material into peritoneal cavity

1. Concomitantly, prepare the recipient mouse for surgery.
2. Maintain sterile technique throughout procedures (see pre-operative care SOP1)
3. Weigh and anaesthetize animals using inhalation anaesthesia (i.e. isoflurane) following appropriate (facility-specific) SOP.
4. Position animal as required in surgical area, on top of a heating pad, disinfect skin by applying disinfectant solution as required by institution-specific protocol (e.g. Povidone-Iodine Swabstick and 70% isopropanol 3 x each ending with isopropanol).
5. Make midline incision in skin using sharp scissors and gently separate skin from underlying fascia.
6. Make a small incision (about 5 mm) in fascia to open abdominal cavity.
7. Hold incision open with forceps while pipet tip is inserted into peritoneal cavity.
8. Inject minced uterine tissue into peritoneal cavity slowly sweeping tissue into the whole cavity in a "J" motion.
9. With forceps, hold peritoneal wall together for 30 seconds.
10. Close skin incision with 9 mm wound clip.
11. Place mouse back into home cage on heating pad.

See SOP10 (post-operative care) for more details.

SOP 7: Endpoint assessment and collection of endometriosis lesions

Purpose

To ensure the maximum amount of data is collected from each animal experiment.

Notes

Endometriosis lesions are usually collected 21 to 40 days post induction, but timing can be altered to fit experimental questions. Other tissues and endpoints are assessed and we recommend collecting as many tissues as possible to facilitate cross collaboration and support future studies as well as reducing the number of animals used.

PROTOCOL

1. For intact mice, we recommend mice be euthanized during the same stage of the oestrous cycle (e.g. oestrus) for direct comparison of gene expression/protein changes across all lesions. Many targets are hormonally responsive and cycle staging provides a similar baseline for comparison of lesions.
2. Mice should be euthanized by an approved/appropriate method and death confirmed (most commonly used method is CO_2 followed by cervical dislocation as confirmation of death).
3. Tissues should be dissected as quickly as possible from the animal, alongside any other tissues of interest for analysis. If storing tissue for RNA or protein, keep on ice and snap freeze as soon as possible.

A. Tissue collection

1. Cycle staging
   a. Critical if examining responses that could be hormonally regulated.
2. Tissue processing
   a. Peritoneal wash: Peritoneal lavage can be recovered by injecting approximately 1 ml buffer to collect cells and for cytokine analysis into the peritoneal cavity followed by gentle massage and recovery. Alternatively, 7 ml of buffer can be used to collect a larger number of cells.
   b. Lesions can be snap frozen for RNA/protein or fixed in neutral buffered formalin or paraformaldehyde for paraffin embedding and histological assessment. Lesions that are cleaned of adjacent tissue can be used for RNA/protein work. Lesions that are removed with adjoining tissue can be embedded *en bloc* for histological orientation and to examine lesion interaction with adjoining tissue. Other organs as per research question (e.g. uterus, ovary, peritoneal tissue biopsy, spleen etc), components of the peripheral (DRG), central nervous system (spinal cord, brain; see Dodds *et al*., 2025), and blood/serum.

B. Endpoint assessment:

1. Imaging
   a. Representative images of lesions *in vivo* and/or *ex vivo*
2. Lesion and pelvic findings
   a. Rodent diagram for recording lesion location (see example below)
   b. Lesion weight and colour
   c. Uterine weight
   d. Ovary weight
   e. Presence of adhesions/adhesion location
   f. Swollen lymph nodes
   g. Lesions attached/unattached
   h. Confirmatory histopathology
3. Fertility
   a. Time to plug

b. Number of pregnant vs non pregnant
c. Embryonic weights
d. Maternal weight
e. Litter size
f. Pup weight
g. Milk spots

4. Pain (see Dodds *et al.*, 2025)

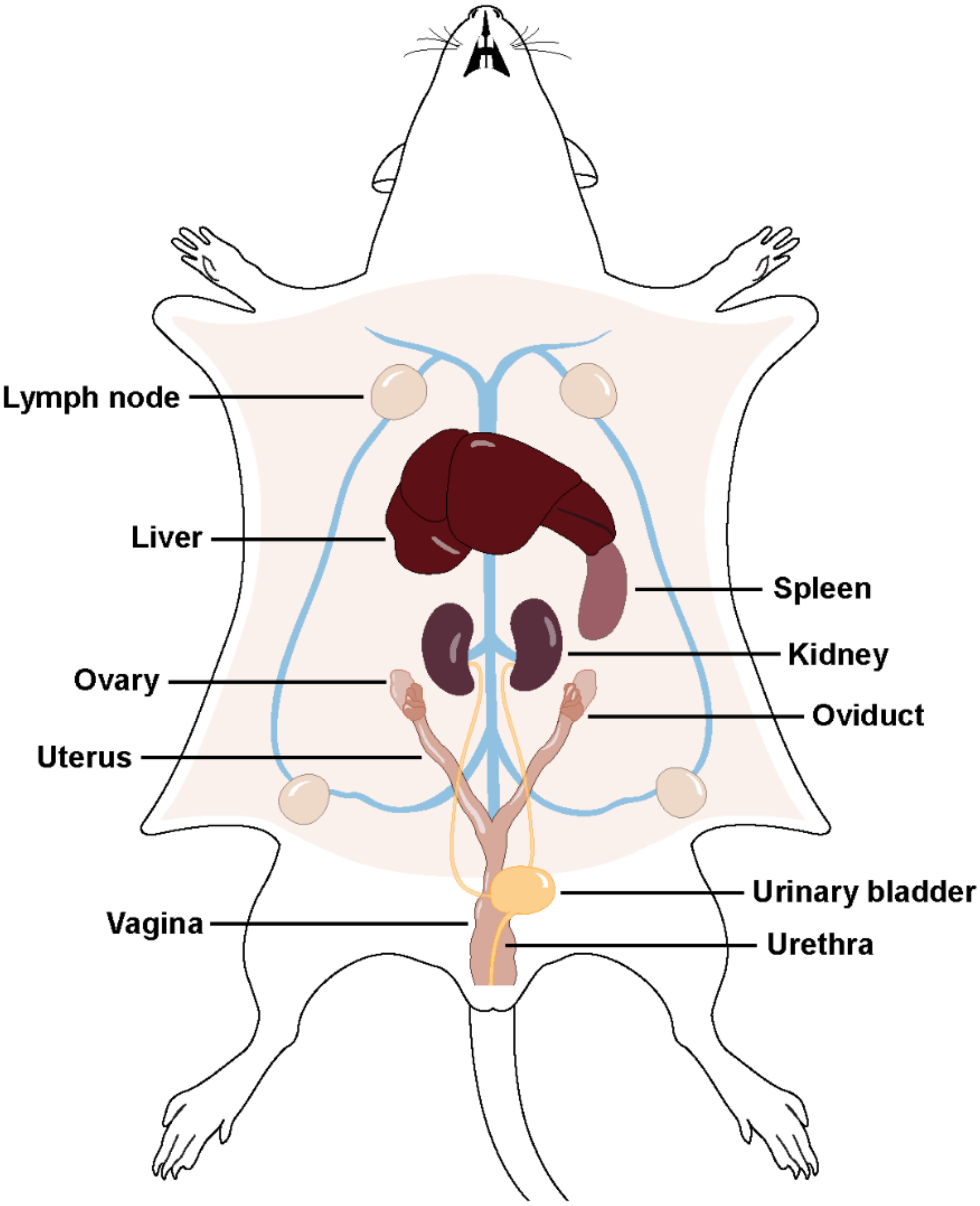

Figure 2. Diagram for use in necropsy to locate endometriotic lesions: *The use of a diagram for each animal will allow for standardization of findings to indicate lesion location, size (weight and volume), colour, etc. The diagram can also be used to note other normal or abnormal findings (e.g. uterine weight, ovary weight, liver appearance).*

SOP 8: Oestrous cycle staging*

Purpose

To establish the stage of oestrous cycle in the mouse.

Notes

Mice cycle through proestrus, oestrus, metestrus, and dioestrus over a 4 - 5 day period (Ajayi and Akhigbe, 2020; Cora *et al.*, 2015). To euthanize mice in a specific cycle stage, mice undergo daily vaginal smears beginning 7 days prior to harvest week. Mice are most frequently euthanized in oestrus, but this may depend on the study objectives.

*If not using filter top cages, male bedding must be added for females to cycle regularly.

PROTOCOL

A. Obtain vaginal cells

1. Manually restrain mice.
2. The tip of a micropipette tip with 50 µl of 0.9% saline is inserted (approximately 2 mm) into vaginal opening and pipetted up and down 2 - 4 times.
3. Vaginal lavage is placed on uncharged slides.
4. Cell populations can be read immediately using the wet preparation to read oestrous cycle stage, typically with an inverted microscope.
5. Lavages on slides can also be left to dry. Slides are sprayed with 100% ethanol to fix cells. Once dry, stain with haematoxylin and eosin to read oestrous cycle stage.

B. Histological examination of vaginal smears

1. Vaginal smears are scored as follows:
   - proestrus (nucleated rounded epithelial cells with few cornified epithelial cells, no leukocytes),
   - oestrus (cornified epithelial cells, strong pink, no leukocytes),
   - metestrus (mostly cornified epithelial cells, few nucleated epithelial cells with few leukocytes),
   - dioestrus (abundant leukocytes, some nucleated epithelial cells and rare cornified cells).
2. Vaginal cytology is read and confirmed by two people blinded to animal treatment.
3. Complete daily until mouse enters oestrus during the harvest week.
4. Mice are sacrificed during oestrus (or consistent cycle stage) to mitigate any confounding factors due to different hormonal changes.

SOP 9: Ovariectomy

Purpose

Mice are ovariectomised in order to ablate the naturally rapidly cycling hormone production and allow 17β-oestradiol to be replaced exogenously.

PROTOCOL

A. **Pre operative preparation** (see SOP1)

B. **Surgical procedure**

1. Make a midline incision in the skin using sharp scissors and gently separate the skin from the underlying fascia.
2. Locate the adipose tissue and ovary position through the fascia and make a 2-4 mm incision in the fascia to open the abdominal cavity.
3. Gently tease the adipose tissue surrounding the ovary through the incision until the ovary is visible.
4. Using forceps, clamp underneath the ovary, cut the ovary free and gently release the ovarian pedicle within the abdominal cavity.
5. Gently ease the adipose tissue back into the body cavity. Incisions less than 1/2 cm will heal without sutures, but larger incisions should be closed using monofilament suture.
6. Make another incision in the contralateral fascia and repeat the ovariectomy procedure.
7. Close the skin wound using wound clips; Wound clips should be removed 7 days after procedure.

C. **Post operative care** (see SOP10)

SOP 10: Post-operative care

1. Place animals on their side or front, in a quiet warm recovery area (30-34°C) until conscious and motor function has returned. Ensure animals have access to water. Monitor respiration. *Animals can be placed in a clean cage lined with a clean paper towel until they have recovered to prevent aspiration of bedding or damage to the eye. Fluid replacement and addition of mash to the cage should be considered and post-operative checks should be undertaken.*
2. Once fully conscious, transfer mice to home cage. Monitor mice daily for signs of pain or distress for 72 hours and give analgesia if necessary and in-line with animal facility guidance.

Standardisation of homologous rat models of experimental endometriosis

Statement of purpose

The purpose of these standard operating procedures (SOPs) is to describe the techniques used to model endometriosis-like lesions in a recipient **rat** via the surgical implantation method. EPHect's purpose in the publication of these procedures is the standardisation of endometriosis experimentation across institutions to create comparable data, ensure experimental integrity, and provide the mechanisms that lead to "best fit models" to recapitulate human disease aetiology.

Criteria

Endometriosis is defined as immune infiltrated ectopic endometrial-like tissue, consisting of stromal cells, glandular epithelial cells, presence of hemosiderin and accumulation of extracellular matrix consistent with fibrosis. For the basis of any current or future model, the endometriosis-like lesions need to meet these criteria with the recognition that lesion histology is incredibly diverse and that on occasions, endometriosis lesions might be harvested that do not contain all above-mentioned hallmarks of endometriotic lesions.

Recommendations

A number of recommendations were made to begin to harmonize how rodent studies on endometriosis are performed and collected. Standards and forms were developed for the model variables (see Rat-Homologous-SOPs below). Overall, most commonly, studies begin with animals in the age range of 8-12 weeks. At surgery, if surgery is performed, most protocols used buprenorphine for pain control. This should be discussed with individual animal units and vets to ensure compliance and mitigate pain in animals, although we recognise that availability in some locations may be limited. Pain management immediately post-surgery is recommended, however we suggest avoiding or minimizing the use of analgesic agents with anti-inflammatory actions (opioids, non-steroidal anti-inflammatory (NSAIDs), and others) to mitigate potential confounding inflammatory processes that are features of endometriosis. When anesthetizing the animals, isoflurane and oxygen is most commonly used with ketamine being used for lengthier surgeries (i.e. laparoscopic).

To standardise studies more formally across various aspects of science and disease, general practices should include power calculations to determine the number of animals needed for proper statistical analysis, randomization of animals and samples when processing, and to follow the Animal Research: Reporting of *In Vivo* Experiments (ARRIVE) guidelines set by the National Centre for the Replacement, Refinement, and Reduction of Animals in Research (Percie du Sert *et al.*, 2020a, Percie du Sert *et al.*, 2020b). All of these general practices strongly complement the recommendations made by WERF for the use of rodent models of endometriosis.

Key model variables and recommendations were categorized for homologous **rat** models of endometriosis as follows:

1. <u>Strain</u>: Sprague-Dawley female rats approximately 8-10 weeks of age (200 ± 10g) at the time of the surgery is the most commonly used strain.
2. <u>Uterine donor tissue 'type'</u>: The aetiology of endometriosis remains unknown; thus, we are unable to make a recommendation of the 'type' of uterine tissue used to establish experimental lesions. Not standardizing this variable preserves the ability of the models to evolve alongside scientific knowledge and advancements. Currently for the rat protocols, 4-6 pieces of full thickness uterus including myometrium are used. This implantation method leads to cysts/vesicles which exhibit similar signs and symptoms to human disease including reduced fertility and increased vaginal hyperalgesia.
3. <u>Method of induction</u>: In rats, the most common method is surgical implantation.
4. <u>Placement of tissue</u>: Peritoneal cavity placement is recommended where uterine tissue is sutured on to the intestinal mesentery.

5. Amount of tissue: Typically, 4-6 pieces of uterus are used.
6. Hormonal status of recipient: Intact rats are recommended.
7. Endpoint assessment and collection of endometriosis lesions: Experimental end points should be hypothesis driven. Collection of multiple endpoint assessments will allow for improved study comparability.

The following SOPs are including in the document

Pre- and post-operative care

- SOP 11: Pre-operative care
- SOP 15: Post-operative care

Procedures for the induction of endometriosis

- SOP 12: Induction of endometriosis using full thickness uterus

Endpoint assessment and collection of endometriosis 'lesions'

- SOP 13: Endpoint assessment and collection of endometriosis lesions

Oestrous Cycle Staging

- SOP 14: Vaginal smears

REFERENCES

Percie du Sert N, Ahluwalia A, Alam S, Avey MT, Baker M, Browne WJ, Clark A, Cuthill IC, Dirnagl U, Emerson M *et al.* Reporting animal research: Explanation and elaboration for the ARRIVE guidelines 2.0. *PLoS Biol* 2020a;18:e3000411.

Percie du Sert N, Hurst V, Ahluwalia A, Alam S, Avey MT, Baker M, Browne WJ, Clark A, Cuthill IC, Dirnagl U *et al.* The ARRIVE guidelines 2.0: Updated guidelines for reporting animal research. *PLoS Biol* 2020b;18:e3000410.

SOP 11: Pre-operative care

Purpose

To prepare rodents for surgery. Prior to conducting any surgical procedures, rats are acclimated (if received from a supplier or other unit), given a health check, and weighed. Surgical days should be scheduled early in the day and conducted at the beginning of the week to allow for adequate monitoring of animals. All instruments should be sterilized by autoclaving before use and can be sterilized between animals using a bead sterilizer or cold sterilant.

PROTOCOL

1. Sterile surgical gloves must be worn. All areas should be cleaned with a sterilizing agent (e.g. Trigene wipes™ or Super Sani-Cloth®) including heating pad/blanket where possible.
2. Place sterile instruments and tools on a sterile surface (e.g. autoclaved metal tray, a sterile drape or towel) during the procedure.
3. Weigh and anaesthetise animal using inhalation or injectable anaesthesia following the appropriate (facility-specific) SOP.
4. Check anaesthetic depth by measuring withdrawal and eye position reflexes.

   Note: *In rodents, withdrawal reflexes are tested by pinching the tail and the hind limbs to demonstrate the reflex is absent or barely detectable. The eye becomes fixed following anaesthetic induction and the blink reflex should be absent. A lubricating eye ointment, like Lacri-lube®, can be placed in the eyes whenever anaesthetics are used to prevent physical damage and dryness.*

5. Following anaesthesia induction, carefully monitor and support body temperature, heart, and respiratory rates.
6. Place rodent on a heating pad for the duration of the procedure.

   Note*: Small animals have a high surface area to body weight ratio and lose heat rapidly. As most anaesthetics depress thermoregulation, hypothermia can develop so rodents should be kept on a heat pad for the duration of the procedure to maintain a body temperature between 35-37°C. Ensure all fluids to be administered are warmed to 35-37°C before administration. Respiratory rate should be maintained at 40-100 breaths per minute (bpm) for rodents.*

7. Remove hair from the abdominal surgical site with clippers (approximately 5 x 7.5 cm area).

   Note: *If possible, animal preparation should be in a different area from the surgical area. Leave sufficient margin to ensure the hair does not enter the wound but avoid over clipping as this will remove insulation. Surgical gloves should be changed or hair removal done by a separate person to maintain sterility.*

8. Once correct anaesthetic depth is achieved, transfer animal to a nose cone for surgery if using inhalation and re-check anaesthetic depth.
9. Analgesia may be given prior to surgery by subcutaneous injection into the interscapular region.

   Note: *Buprenorphine (an opioid derivative) is recommended for pain relief Buprenorphine should be administered at a dose of 0.01–0.05 mg/kg.*

10. Position animal as required in the surgical area, on top of a heating pad and disinfect the skin by applying a disinfectant solution (e.g. Hibitane). Apply a suitable drape.

SOP 12: Induction of endometriosis using full thickness uterus

Purpose

A prevailing hypothesis for the development of endometriotic lesions in women is via retrograde menstruation. During menstruation, the endometrium of the uterus is shed. Rats do not menstruate but exhibit hormonal patterns similar to humans in a much shorter period (i.e. 4-5 days). SOP 12 describes the method used by researchers to prepare full thickness uterus for implantation onto the intestinal mesentery in the peritoneal cavity of the rat.

Procedures thoroughly described in multiple references (Berkley *et al.*, 2007; Birt *et al.*, 2013; Cox *et al.*, 2001; Cuevas *et al.*, 2012; Flores *et al.*, 2007; Ingelmo *et al.*, 1999; McAllister *et al.*, 2012; McAllister *et al.*, 2016; McAllister *et al.*, 2009; McAllister *et al.*, 2021; Sharpe-Timms, 2002; Sharpe-Timms *et al.*, 2020; Sharpe-Timms *et al.*, 1998; Sharpe *et al.*, 1991; Sharpe *et al.*, 1990; Stilley and Sharpe-Timms, 2012; Stilley *et al.*, 2009; Vernon and Wilson, 1985; Wright and Sharpe-Timms, 1995).

PROTOCOL

1. Under sterile conditions, perform midline abdominal incision (<2.5 cm) using a scalpel to enter the abdominal cavity.
2. Locate and expose the right uterine horn with the associated ovary.
3. Gently clamp off the middle (approximately 1 cm) segment of the right uterine horn with two haemostats.
4. Ablate the distal 1 cm of uterine horn and place into warm (37°C) sterile saline or RPMI. In some cases, sutures are placed around the uterine horn and arterial cascades to prevent bleeding prior to removal of uterine tissue.
5. Remove excess fat from the excised uterine horn.
6. Cut the uterine horn lengthwise to expose the endometrium and then cut into 4-6 equal 2 x 2 mm pieces.
7. Unclamp the previously placed haemostats and confirm there is no bleeding.
8. Locate the caecum and adjacent small intestines and mesentery within the abdominal cavity using sterile saline-soaked cotton tipped applicators.
9. Use non-absorbable silk or nylon suture (4-0 braided*) and a needle holder to sew one 2 x 2 mm piece of uterus** onto alternate cascading mesenteric arteries that supply the caudal small intestines starting at the caecum.

   Note: **Non-braided suture may cause less bleeding. **Uterine pieces must be sewn with the outer surface next to the mesenteric vessels and the endometrial surface exposed to the peritoneal environment*.

10. Confirm there is no bleeding.
11. Use vicryl absorbable suture (5-0 braided*) and a needle holder to close the muscle layer with a simple interrupted pattern.

    Note: **Non-braided suture may cause less bleeding.*

12. Use 4-0 non-absorbable* monofilament suture and a needle holder to close the skin wound with a simple interrupted pattern.

    Note: **Absorbable sutures are also used.*

13. For sham animals: massage the uteri for 2 minutes with fingertips, then attach 4-0 silk sutures to the mesenteries but without explants.
14. Keep peritoneal cavity moist with copious amounts (i.e. >1 mL) of 0.9% sterile saline during surgery.

15. Confirm lack of bleeding.
16. Close the abdominal cavity with absorbable sutures (5-0 chromic gut suture) in the fascia and non-absorbable sutures in the skin (silk 4-0) in a simple interrupted pattern.

REFERENCES

Berkley KJ, McAllister SL, Accius BE, Winnard KP. Endometriosis-induced vaginal hyperalgesia in the rat: effect of estropause, ovariectomy, and estradiol replacement. *Pain* 2007;132 Suppl 1:S150-S159.

Birt JA, Taylor KH, Davis JW, Sharpe-Timms KL. Developmental exposure of fetal ovaries and fetal germ cells to endometriosis in an endometriosis model causes differential gene expression in the preimplantation embryos of the first-generation and second-generation embryos. *Fertil Steril* 2013;100:1436-1443.

Cox KE, Piva M, Sharpe-Timms KL. Differential regulation of matrix metalloproteinase-3 gene expression in endometriotic lesions compared with endometrium. *Biol Reprod* 2001;65:1297-1303.

Cuevas M, Flores I, Thompson KJ, Ramos-Ortolaza DL, Torres-Reveron A, Appleyard CB. Stress exacerbates endometriosis manifestations and inflammatory parameters in an animal model. *Reprod Sci* 2012;19:851-862.

Flores I, Rivera E, Ruiz LA, Santiago OI, Vernon MW, Appleyard CB. Molecular profiling of experimental endometriosis identified gene expression patterns in common with human disease. *Fertil Steril* 2007;87:1180-1199.

Ingelmo JM, Quereda F, Acien P. Intraperitoneal and subcutaneous treatment of experimental endometriosis with recombinant human interferon-alpha-2b in a murine model. *Fertil Steril* 1999;71:907-911.

McAllister SL, Dmitrieva N, Berkley KJ. Sprouted innervation into uterine transplants contributes to the development of hyperalgesia in a rat model of endometriosis. *PLoS One* 2012;7:e31758.

McAllister SL, Giourgas BK, Faircloth EK, Leishman E, Bradshaw HB, Gross ER. Prostaglandin levels, vaginal innervation, and cyst innervation as peripheral contributors to endometriosis-associated vaginal hyperalgesia in rodents. *Mol Cell Endocrinol* 2016;437:120-129.

McAllister SL, McGinty KA, Resuehr D, Berkley KJ. Endometriosis-induced vaginal hyperalgesia in the rat: role of the ectopic growths and their innervation. *Pain* 2009;147:255-264.

McAllister SL, Sinharoy P, Vasu M, Gross ER. Aberrant reactive aldehyde detoxification by aldehyde dehydrogenase-2 influences endometriosis development and pain-associated behaviors. *Pain* 2021;162:71-83.

Sharpe-Timms KL. Using rats as a research model for the study of endometriosis. *Ann N Y Acad Sci* 2002;955:318-327; discussion 340-312, 396-406.

Sharpe-Timms KL, Nabli H, Stilley JAW. Identifying Mechanisms of Endometriosis-Associated Reduced Fecundity in a Rat Model: Novel Insights toward Understanding Human Infertility. *Adv Anat Embryol Cell Biol* 2020;232:9-24.

Sharpe-Timms KL, Zimmer RL, Jolliff WJ, Wright JA, Nothnick WB, Curry TE. Gonadotropin-releasing hormone agonist (GnRH-a) therapy alters activity of plasminogen activators, matrix metalloproteinases, and their inhibitors in rat models for adhesion formation and endometriosis: potential GnRH-a-regulated mechanisms reducing adhesion formation. *Fertil Steril* 1998;69:916-923.

Sharpe KL, Bertero MC, Muse KN, Vernon MW. Spontaneous and steroid-induced recurrence of endometriosis after suppression by a gonadotropin-releasing hormone antagonist in the rat. *Am J Obstet Gynecol* 1991;164:187-194.

Sharpe KL, Bertero MC, Vernon MW. Rapid regression of endometriosis by a new gonadotropin-releasing hormone antagonist in rats with surgically induced disease. *Prog Clin Biol Res* 1990;323:449-458.

Stilley JA, Sharpe-Timms KL. TIMP1 contributes to ovarian anomalies in both an MMP-dependent and -independent manner in a rat model. *Biol Reprod* 2012;86:47.

Stilley JA, Woods-Marshall R, Sutovsky M, Sutovsky P, Sharpe-Timms KL. Reduced fecundity in female rats with surgically induced endometriosis and in their daughters: a potential role for tissue inhibitors of metalloproteinase 1. *Biol Reprod* 2009;80:649-656.

Vernon MW, Wilson EA. Studies on the surgical induction of endometriosis in the rat. *Fertil Steril* 1985;44:684-694.

Wright JA, Sharpe-Timms KL. Gonadotropin-releasing hormone agonist therapy reduces postoperative adhesion formation and reformation after adhesiolysis in rat models for adhesion formation and endometriosis. *Fertil Steril* 1995;63:1094-1100.

SOP 13: Endpoint assessment and collection of endometriosis lesions

Purpose
To ensure the maximum amount of data is collected from each animal experiment.

Notes
Endometriosis lesions in the rat become vascularized and grow rapidly in the first month, stabilize by 2 months, and remain viable >10 months. Lesions are typically collected dependent on the experimental outcomes (i.e. 7 days, 14 days, 21 days, and 60 days). Other tissues and endpoints are assessed, and we recommend collecting as many tissues as possible to facilitate cross collaboration and support future studies as well as reducing the number of animals used.

For rats, we recommend they be euthanized during the same stage of the oestrous cycle (e.g. oestrus) for direct comparison of gene expression/protein changes across all lesions. However, for longer duration behavioural/intervention experiments animals may be sacrificed on a predesignated number of days after induction surgery. Many targets are hormonally responsive and cycle staging provides a similar baseline for comparison of lesions.

PROTOCOL
1. Rats should be euthanized by an approved/appropriate method and death confirmed (most commonly used method is a lethal dose of pentobarbital).
2. Tissues should be dissected as quickly as possible from the animal, alongside any other tissues of interest for analysis. If storing tissue for RNA or protein, keep on ice and snap freeze as soon as possible.

A. Tissue collection

1. Cycle staging
    a. Critical if examining responses that could be hormonally regulated.
2. Tissue processing
    a. Peritoneal fluid: after making a central midline incision in the rat's abdomen, collect peritoneal fluid with sterile transfer pipet.
    b. To collect blood, perform cardiac puncture.
    c. Lesions can be snap frozen for RNA/protein or fixed in neutral buffered formalin or paraformaldehyde for paraffin embedding and histological assessment. Lesions that are cleaned of adjacent tissue can be used for RNA/protein work. Lesions that are removed with adjoining tissue can be embedded *en bloc* for histological orientation and to examine lesion interaction with adjoining tissue. Other organs as per research question (e.g. uterus, ovary, peritoneal tissue biopsy, spleen etc), components of the peripheral (DRG), central nervous system (spinal cord, brain; see Dodds *et al*., tbc), and blood/serum.

B. Endpoint assessment

1. Imaging
    a. Representative images of lesions *in vivo* and/or *ex vivo*
2. Lesion and pelvic findings
    a. Diagram for recording lesion location (See Figure 2)
    b. Lesion weight, size, and colour
    c. Uterine weight
    d. Ovary weight

   e. Presence of adhesions/adhesion location
   f. Swollen lymph nodes
   g. Lesions attached/unattached
   h. Confirmatory histopathology
3. Fertility
   a. Dependent on study endpoint and question
   b. More reproducible in rats than mice (possibly due to limited studies).
4. Pain (see Dodds *et al.*, 2025)

SOP 14: Oestrous cycle staging*

Purpose
To establish the stage of oestrous in the rat.

Notes
Rats cycle through proestrus, oestrus, metestrus, and dioestrus over a 4 - 5 day period (Ajayi and Akhigbe, 2020; Cora *et al.*, 2015). Rat vaginal cytology is evaluated daily using the (swab or smear) technique beginning 7 days prior to surgery to ensure normal cycling and maturity, as well as after surgery to ensure continuation of normal cycling. Daily animal handling helps to decrease experimenter-induced stress. Also used to determine day of induction if surgery is to be carried out on a specific stage of the cycle (e.g. oestrus) or used to determine day of harvest if necropsy is to be carried out on a specific stage of the cycle.

PROTOCOL

A. Obtain vaginal swab or smear

1. Manually restrain rat.
2. The tip of a micropipette tip with 500 µl of 0.9% saline is inserted (approximately 0.5-1 cm) into vaginal opening and pipetted up and down 2 - 4 times. *OR* using a swab stick dipped into 0.9% saline, gently swab for a full turn the vaginal opening.
3. Vaginal wash or swab tip contents are placed on uncharged slides and allowed to completely dry.
4. Slides are sprayed with 100% ethanol to fix cells. Once dry, stain with haematoxylin and eosin or Wright's stain to read oestrous cycle stage.

B. Histological examination of vaginal smears

1. Vaginal smears are scored as follows:
   - proestrus (nucleated epithelial cells with no or few cornified epithelial cells),
   - oestrus (cornified cells, strong pink),
   - metestrus (epithelial cells with leukocyte attacks),
   - dioestrus (few leukocytes and/or cornified cells).

2. Vaginal cytology is read and confirmed by two people blinded to animal treatment.
3. Complete daily until rat enters desired stage.
4. Rats can be cycle staged for sacrifice to mitigate any confounding factors due to different hormonal changes if experimental endpoints warrant staging.

REFERENCES

Ajayi AF, Akhigbe RE. Staging of the estrous cycle and induction of estrus in experimental rodents: an update. *Fertil Res Pract* 2020;6:5.

Cora MC, Kooistra L, Travlos G. Vaginal Cytology of the Laboratory Rat and Mouse: Review and Criteria for the Staging of the Estrous Cycle Using Stained Vaginal Smears. *Toxicol Pathol* 2015;43:776-793.

SOP 15: Post-operative care

1. Place animals on their side, in a quiet warm recovery area (30 - 34°C) until conscious and motor function has returned. Ensure animals have access to water. Monitor respiration. *Animals can be placed in a clean cage lined with a clean paper towel until they have recovered to prevent aspiration of bedding or damage to the eye. Fluid replacement and addition of mash to the cage should be considered and post-operative checks should be undertaken.*

2. Once fully conscious, transfer rats to home cage. Monitor rats daily for signs of pain or distress for 72 hours and give analgesia if necessary and in-line with respective animal facility guidance.
